# *JAK2* aberrations in childhood B-cell precursor acute lymphoblastic leukemia

**DOI:** 10.18632/oncotarget.21027

**Published:** 2017-09-16

**Authors:** Elisabeth M.P. Steeghs, Isabel S. Jerchel, Willemieke de Goffau-Nobel, Alex Q. Hoogkamer, Judith M. Boer, Aurélie Boeree, Cesca van de Ven, Marco J. Koudijs, Nicolle J.M. Besselink, Hester A. de Groot-Kruseman, Christian Michel Zwaan, Martin A. Horstmann, Rob Pieters, Monique L. den Boer

**Affiliations:** ^1^ Department of Pediatric Oncology/Hematology, Erasmus Medical Centre – Sophia Children's Hospital, Rotterdam, The Netherlands; ^2^ Centre for Personalized Cancer Treatment, Utrecht, The Netherlands; ^3^ Department of Medical Genetics, University Medical Centre, Utrecht, The Netherlands; ^4^ DCOG, Dutch Childhood Oncology Group, The Hague, The Netherlands; ^5^ COALL - German Cooperative Study Group for Childhood Acute Lymphoblastic Leukemia, Hamburg, Germany; ^6^ Princess Máxima Centre for Pediatric Oncology, Utrecht, The Netherlands

**Keywords:** JAK2, B-cell precursor acute lymphoblastic leukemia, pediatric, targeted therapies, JAK inhibitors

## Abstract

*JAK2* abnormalities may serve as target for precision medicines in pediatric B-cell precursor acute lymphoblastic leukemia (BCP-ALL). In the current study we performed a screening for *JAK2* mutations and translocations, analyzed the clinical outcome and studied the efficacy of two JAK inhibitors in primary BCP-ALL cells. Importantly, we identify a number of limitations of JAK inhibitor therapy.

*JAK2* mutations mainly occurred in the poor prognostic subtypes *BCR-ABL1*-like and non- *BCR-ABL1*-like B-other (negative for sentinel cytogenetic lesions). *JAK2* translocations were restricted to *BCR-ABL1*-like cases. Momelotinib and ruxolitinib were cytotoxic in both *JAK2* translocated and *JAK2* mutated cells, although efficacy in *JAK2* mutated cells highly depended on cytokine receptor activation by TSLP. However, our data also suggest that the effect of JAK inhibition may be compromised by mutations in alternative survival pathways and microenvironment-induced resistance. Furthermore, inhibitors induced accumulation of phosphorylated JAK2^Y1007^, which resulted in a profound re-activation of JAK2 signaling upon release of the inhibitors. This preclinical evidence implies that further optimization and evaluation of JAK inhibitor treatment is necessary prior to its clinical integration in pediatric BCP-ALL.

## INTRODUCTION

Janus kinase 2 (JAK2) is a member of the non-receptor tyrosine kinase family and mediates intracellular signaling upon activation of cytokine receptors, which lack an intrinsic tyrosine kinase domain, such as cytokine receptor-like factor 2 (CRLF2). Ligand binding (e.g. TSLP for CLRF2) induces dimerization of cytokine receptors chains, resulting in activation of JAK proteins via cross-phosphorylation. JAKs activate signal transducers of transcription (STATs), which, upon dimerization, migrate to the nucleus and induce transcription of genes involved in differentiation and proliferation of hematopoietic cells [1].

JAK2 has seven homologous domains (JH1-JH7). The JH1 and JH2 domains are C-terminally located and comprise the catalytic kinase (JH1) and pseudokinase (JH2) domain. The JH2 domain has a dual regulatory function: exerting a negative regulatory effect on the kinase domain, and facilitating JAK2 activation upon receptor activation by ligand binding [2]. The JH3-JH4 domains share homology with Src homology 2 (SH2) domains and mediate protein-protein interactions. The N-terminal located JH6 and JH7 domains, also known as the FERM domain, are required for binding of JAK2 to cytokine receptors [1].

In pediatric BCP-ALL patients, gain of function mutations and translocations affecting *JAK2* have been identified [3–16]. Genomic translocations of *JAK2* have been observed in high-risk *BCR-ABL1*-like patients. For several of these fusion genes, constitutive JAK2 kinase activation has been demonstrated [12–14, 17]. Point mutations often occur in Down Syndrome ALL, mainly affect exon 16 (located in the pseudokinase domain), and functionally cooperate with overexpression of the type I cytokine receptor *CRLF2* [6, 9, 10]. Indeed, requirement for the interaction of mutant *JAK2* with a cytokine receptor was shown in cell lines models by several groups [6, 8, 9].

Mutations and translocations represent biologically distinct entities, but both are potential targets for precision medicines. JAK inhibitors were shown to be effective against mutant and translocated *JAK2 in vitro* [3, 5, 7, 8, 12, 13, 17, 18]. However, *in vivo* mouse studies show conflicting data and none has been reported to be curative [13, 18–22]. To date, clinical data with JAK inhibitors are scarce. The Children's Oncology Group performed a phase 1 dosing study of the JAK inhibitor ruxolitinib, but no cases harboring *JAK2* activating mutations or translocations were included [23].

Several papers have reported data with a focus on either fusion genes or mutations of *JAK2*, although often with small sample size or only in specific subtypes of BCP-ALL. Furthermore, most reports lack *ex vivo* efficacy data of JAK inhibitors in primary leukemic cells. To assess the clinical potential of JAK inhibitors in pediatric BCP-ALL, we performed a comprehensive study to determine the frequency and prognosis of *JAK2* mutations and translocations among different subtypes of childhood BCP-ALL. Furthermore, the biological efficacy of the JAK inhibitors momelotinib and ruxolitinib was studied in primary leukemic cells harboring *JAK2* aberrations, and the clonal stability of *JAK2* mutations was investigated in ALL patient derived xenograft models. We show that JAK inhibitors are overall effective towards BCP-ALL cells, but also identified a number of limitations of JAK inhibitor therapy.

## RESULTS

### Frequency and type of JAK2 aberrations in BCP-ALL patients

**Figure 1:**
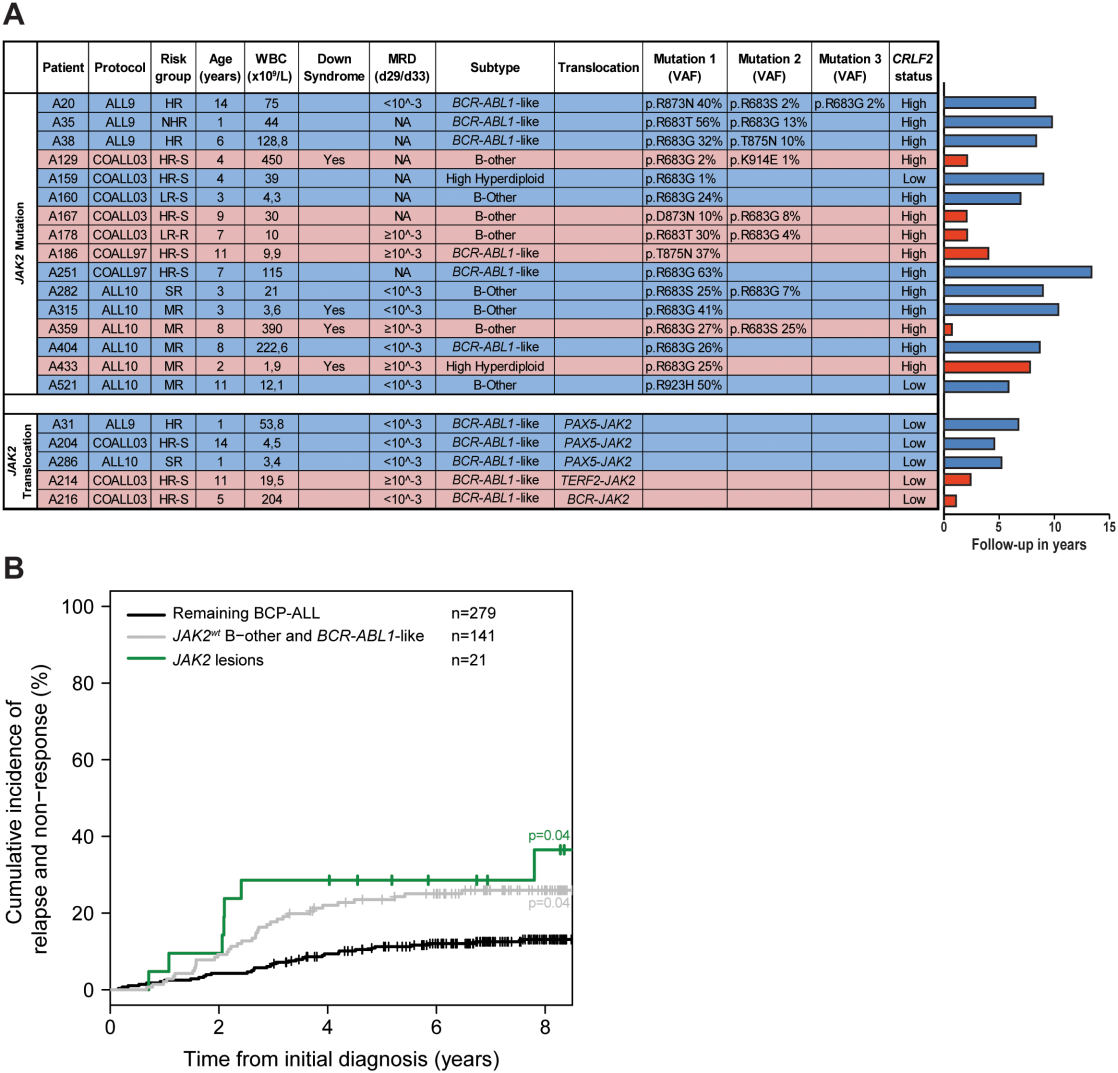
JAK2 aberrations in BCP-ALL patients **(A)** Type of lesions, clinical characteristics and follow-up of *JAK2* lesion positive patients. Treatment protocol and risk group assigned to each patient per protocol have been listed (HR-S: High Risk Standard. HR: High Risk. MR: Median Risk. SR: Standard Risk. LR-S: Low Risk Standard. LR-R: Low Risk Reduced. NHR: Non-High Risk). WBC indicates white blood cell count. Minimal residual disease (MRD) levels at day 29/33 of treatment of COALL and DCOG protocol, respectively. Type of translocation or mutation is listed. VAF indicates the variant allele frequency (%). CRLF2 status indicates gene expression below (low) or above (high) the 90^th^ percentile levels. Right panel: Bar plot represents years from diagnosis to event or last contact. In blue: cases in complete clinical remission. In red: cases with an event (relapse or death). **(B)** Cumulative incidence of relapse curves for patients with *JAK2* lesions (green line), *JAK2* wildtype *BCR-ABL1*-like and B-other cases (grey line), and *JAK2* wildtype remaining BCP-ALL cases (black line; *ETV6-RUNX1*, high hyperdiploid, *TCF3-PBX1*). Patients were treated according to ALL8, ALL9, ALL10, COALL97 or COALL03 protocol. Cumulative incidence of relapse (CIR) was estimated using a competing risk model. Relapse and non-response were considered as event, and death as competing event. Non-response was counted as event at day 79. The Gray's test was applied to test for equality of CIRs (*JAK2* lesion versus remaining BCP-ALL p=0.04; *JAK2* wildtype B-other/*BCR-ABL1*-like versus remaining BCP-ALL p=0.04).

*JAK2* mutation status was analyzed in 461 newly diagnosed BCP-ALL cases representing all major subtypes seen in children, with a distribution that is comparable to the general pediatric BCP-ALL population. *JAK2* exons 16, 20, 21 and 23 were examined by targeted amplicon sequencing at a median read depth of 673, 577, 711 and 944, respectively. Analyses revealed that 3.5% (16/461) of these BCP-ALL cases harbored *JAK2* mutations, which were detected in 7.6% (6/79) of *BCR-ABL1*-like cases, 11.9% (8/67) of non-*BCR-ABL1*-like B-other cases, and 1.6% (2/124) of high hyperdiploid cases. No *JAK2* mutations were detected in *KMT2A*-*AFF1* (0/15), *BCR-ABL1* (0/26), *ETV6-RUNX1* (0/124) or *TCF3-PBX1* (0/26) cases. The variant allele frequency (VAF) ranged from 1.0% to 56% (Figure 1A). Seven patients carried two different *JAK2* mutations, and one patient even harbored three different *JAK2* mutations. Mutations involved amino acid residue R683 in 13 of 16 mutated cases, which is an important amino acid for the JH2 domain mediated negative auto-regulation of JAK2 activity [24]. *CRLF2* overexpression was detected in 87.5% (14/16) of these cases (Figure 1A, Supplementary Figure 1). One *CRLF2* low expressing case harbored a subclonal *JAK2* mutation, suggesting that *CRLF2* overexpression might be subclonal as well. The other case harbored a *JAK2^R923H^* with a VAF of 50%, suggesting that this mutation in the kinase domain is not associated with *CRLF2* overexpression.

The screen for *JAK2* fusion genes was confined to 153 BCP-ALL cases, negative for sentinel BCP-ALL associated lesions (*KMT2A*-rearranged, *BCR-ABL1*, *ETV6-RUNX1*, *TCF-PBX1*, high hyperdiploid), as *JAK2* translocations were previously reported in this group of patients [12, 13, 25]. No *JAK2* translocations were detected in 76 non-*BCR-ABL1*-like B-other cases, whereas in five of the 77 (6.5%) *BCR-ABL1*-like cases *JAK2* tyrosine kinase activating fusion genes were identified. The cases involved three *PAX5-JAK2* cases, one *BCR-JAK2* case and one *TERF2-JAK2* case (Figure 1A). The *PAX5-JAK2* and *BCR-JAK2* fusions contained identical exons as reported before [12, 13]. The *TERF2-JAK2* case displayed an in frame fusion of *TERF2* exon 10 to *JAK2* exon 19. All *JAK2* fusion genes harbored an intact JH1 kinase domain (Supplementary Figure 2). Gene expression data revealed high expression levels of *JAK2* in these cases (Supplementary Figure 3). Absence of the cytokine receptor-binding FERM domain in JAK2 fusion protein suggests that they signal independent of a cytokine receptor.

### Clinical characteristics and prognosis of patients harboring JAK2 lesions

Ten out of sixteen (62.5%) *JAK2*-mutated patients remained in continuous complete remission at more than 5 years of follow up. The median time to relapse in the six other patients was 2.1 years [range 0.71-7.8 years]. Minimal residual disease (MRD) data were available for nine patients. The four patients with high MRD levels (≥10^-3^) at day 29/33 of treatment (time point 1 of COALL and DCOG protocol, respectively) relapsed, whereas the remaining five mutated patients with low MRD levels remained in continuous complete remission (p=0.008, Fisher exact test).

Three out of the five cases harboring *JAK2* fusion genes remained in continuous complete remission at more than 5 years of follow up, whereas two cases suffered from a relapse within 2.4 years of diagnosis (Figure 1A). Both patients who relapsed were assigned to the High Risk arm of the COALL-03 study protocol because of unfavorable age (>10 years) or high white blood cell count at diagnosis (>50 WBC/nl).

Cumulative incidence of relapse in these *JAK2* aberrant patients did not differ from *JAK2* wildtype *BCR-ABL1*-like and B-other cases. Both displayed an unfavorable outcome compared to remaining BCP-ALL cases (p=0.04; Figure 1B). These findings underline the clinical relevance of *JAK2* lesions. Mutations and translocations represent biologically distinct entities, but both may be targetable by JAK-inhibitors.

### Leukemic cells with JAK2 lesions can be targeted by JAK inhibitors

**Figure 2:**
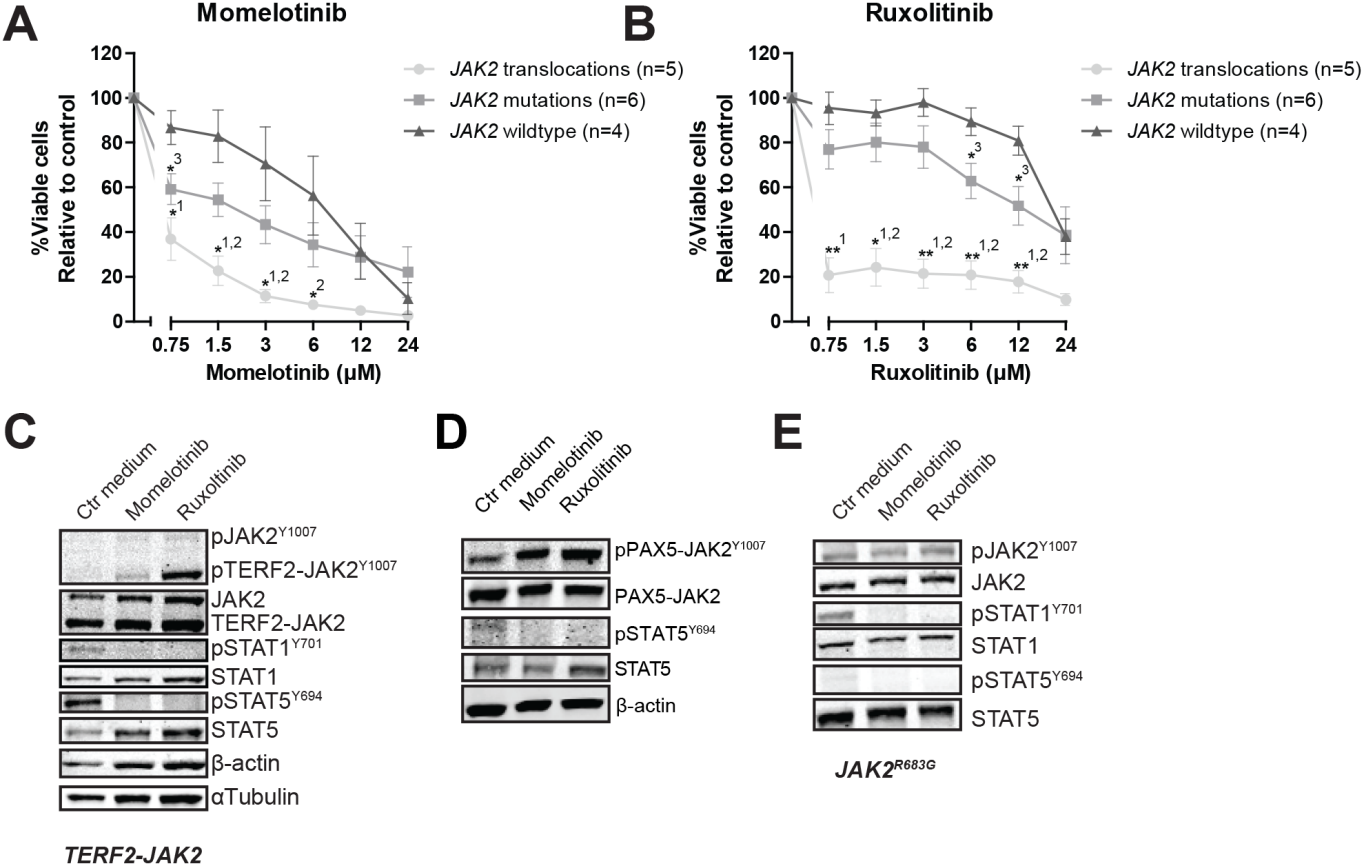
The efficacy of JAK inhibitors on JAK2 translocated and mutated cells **(A-B)** Leukemic (PDX or primary patient) cells were incubated for four days with to an increasing concentration range of momelotinib or ruxolitinib, after which cell viability was measured using an MTT assay. Sensitivity of exposed cells was calculated relative to vehicle treated controls. Individual samples were tested in duplicate. Mean±SEM of *JAK2* translocated cases (n=5), *JAK2* mutated cases (n=6) and *JAK2* wildtype cases (n=4) is shown. Cell viability of samples was compared using independent sample T-test. ^**^p≤0.01, ^*^p≤0.05, ^1^*JAK2* translocations versus *JAK2* wildtype,^2^*JAK2* translocations versus *JAK2* mutations,^3^*JAK2* mutations versus *JAK2* wildtype. **(C-E)** *TERF2-JAK2, PAX5-JAK2* and *JAK2^R683G^* PDX cells were exposed for four hours to vehicle control medium, 1.5 μM momelotinib or 0.75 μM ruxolitinib, after which (phosphorylated) TERF2-JAK2, JAK2, STAT1 and STAT5 levels were analyzed using western blot (25 μg lysate).

Primary leukemic and patient-derived-xenograft (PDX) cells (Supplementary Figure 4, [26]) were exposed to momelotinib and ruxolitinib. *JAK2* translocated cells were more sensitive to both momelotinib and ruxolitinib compared to *JAK2* wildtype cases (P<0.05; Figure 2A and 2B, Supplementary Figure 5. *JAK2* mutated cells were less sensitive to these inhibitors than *JAK2* fusion positive cells, and were only marginally more sensitive than wildtype cells (P<0.05). Leukemic cells without genetic *JAK2* aberrations were resistant to ruxolitinib, but showed some sensitivity to momelotinib. Importantly, normal bone marrow mononuclear cells were resistant to both inhibitors (Supplementary Figure 5). Both JAK inhibitors effectively reduced levels of phosphorylated STAT5^Y694^ and/or STAT1^Y701^ (Figure 2C and 2E, Supplementary Figure 6).

**Figure 3:**
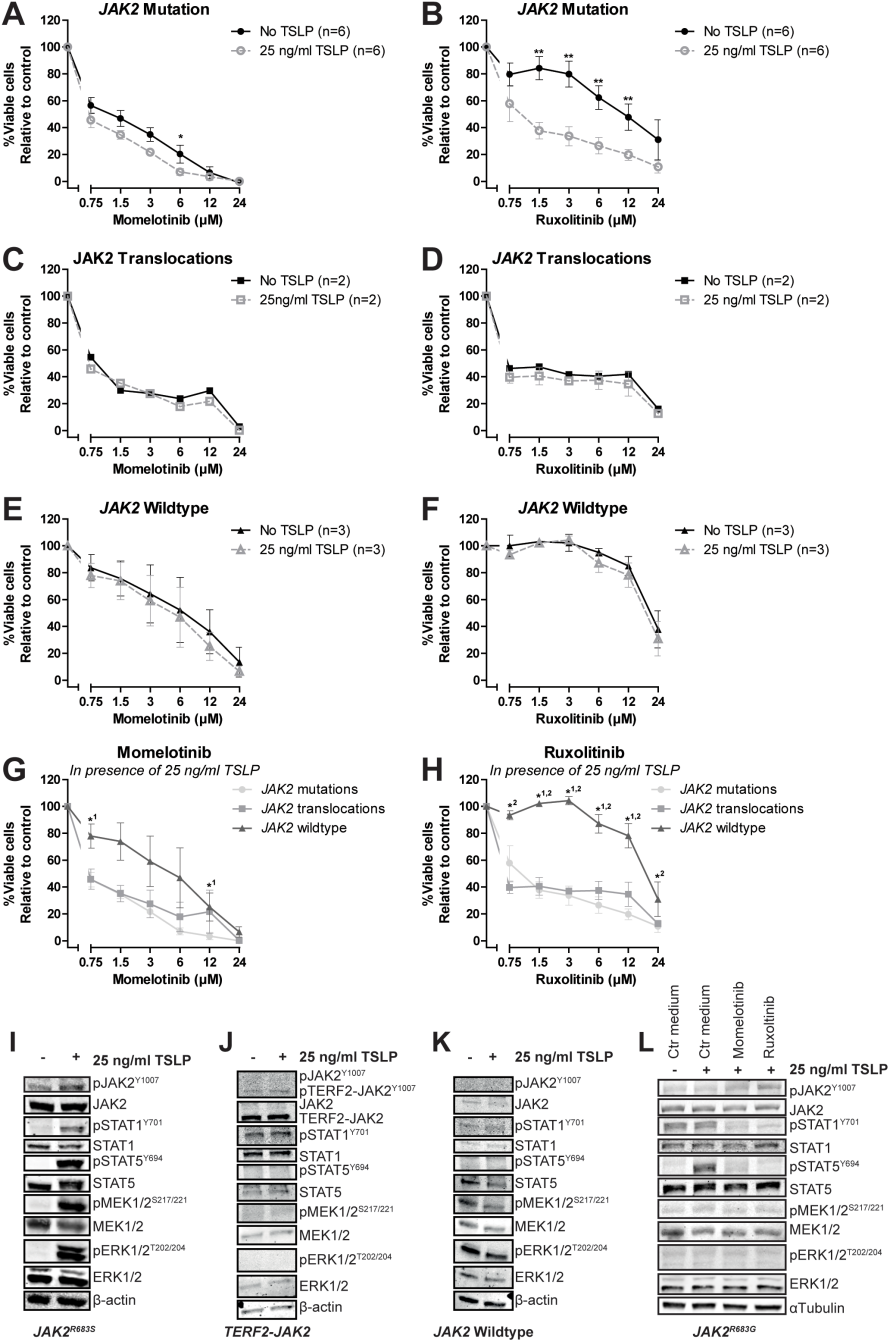
The effect of TSLP stimulation on the efficacy of JAK inhibitors Cells (PDX or primary ALL) were pre-incubated for 1 hour with or without 25 ng/ml TSLP, after which cells were exposed for four days to indicated concentrations of momelotinib or ruxolitinib. Cell viability was measured using an MTT assay. Sensitivity was calculated relative to vehicle treated controls. Individual samples were tested in duplicate. **(A-B)** Efficacy of momelotinib and ruxolitinib on *JAK2* mutated cells with or without TSLP pre-incubation. Mean±SEM of six independent samples is shown. **(C-D)** Efficacy of momelotinib and ruxolitinib on cells with *JAK2* translocations. Mean±SEM of two independent samples is shown. **(E-F)** Efficacy of momelotinib and ruxolitinib on *JAK2* wildtype PDX cells. Mean±SEM of three independent samples is shown. **(G-H)** Combined graph of the efficacy of momelotinib (G) and ruxolitinib (H) on TSLP stimulated cells with *JAK2* mutations (n=6), *JAK2* translocations (n=2), or *JAK2* wildtype cells (n=3). Mean±SEM of independent samples is shown. Cell viability of samples was compared using the independent sample T-test. ^**^p≤0.01, ^*^p≤0.05. ^1^*JAK2* translocations versus *JAK2* wildtype,^2^*JAK2* wildtype versus *JAK2* mutations. **(I-K)** Western blot of *JAK2^R683S^*, *TERF2-JAK2* and *JAK2^wt^* PDX cells with or without TSLP stimulation (25 ng/ml for 1 hour). **(L)** *JAK2^R683G^* cells were pre-incubated for 1 hour with or without 25 ng/ml TSLP, after which cells were exposed for four hours to vehicle control medium, 1.5 μM momelotinib or 0.75 μM ruxolitinib. Levels of (phosphorylated) JAK2, STAT1, STAT5, MEK1/2 and ERK1/2 were analyzed using western blot.

The marginal sensitivity for both inhibitors and the low levels of phosphorylated STAT5 in *JAK2* mutated cells may be explained by lack of human TSLP ligand to activate the CRLF2 pathway in our culture conditions. Addition of human TSLP sensitized *JAK2* mutated cells to ruxolitinib, but not to momelotinib (P<0.01, Figure 3A and 3B, Supplementary Figures 7 and 8). TSLP exposure did not change the efficacy of JAK inhibitors in *JAK2*-fusion positive cells, confirming cytokine-independent signaling (Figure 3C and 3D, Supplementary Figure 7). *JAK2* wildtype leukemic cells were not sensitized to JAK inhibitors by TSLP treatment (Figure 3E and 3F, Supplementary Figure 7). In the presence of TSLP both *JAK2* translocated and *JAK2* mutated cells were sensitive to JAK inhibitors (Figure 3G and 3H). At the protein level, TSLP exposure upregulated the levels of phosphorylated STAT1^Y701^ and STAT5^Y694^ in *JAK2^R683S^* mutated cells, whereas no effect was observed in *JAK2* fusion positive and *JAK2* wildtype leukemic cells (Figure 3I-3K, Supplementary Figure 9). Notably, TSLP triggered the phosphorylation and hence activation of the MEK/ERK pathway in *JAK2^R683S^* mutated cells, but not in *JAK2^R683G^* mutated cells (Figure 3I and 3L), suggesting that this activation is context-dependent. Phosphorylation of STAT1^Y701^ and STAT5^Y694^ was inhibited by momelotinib and ruxolitinib (Figure 3L).

### JAK2 inhibition results in accumulation of phosphorylated JAK2

**Figure 4:**
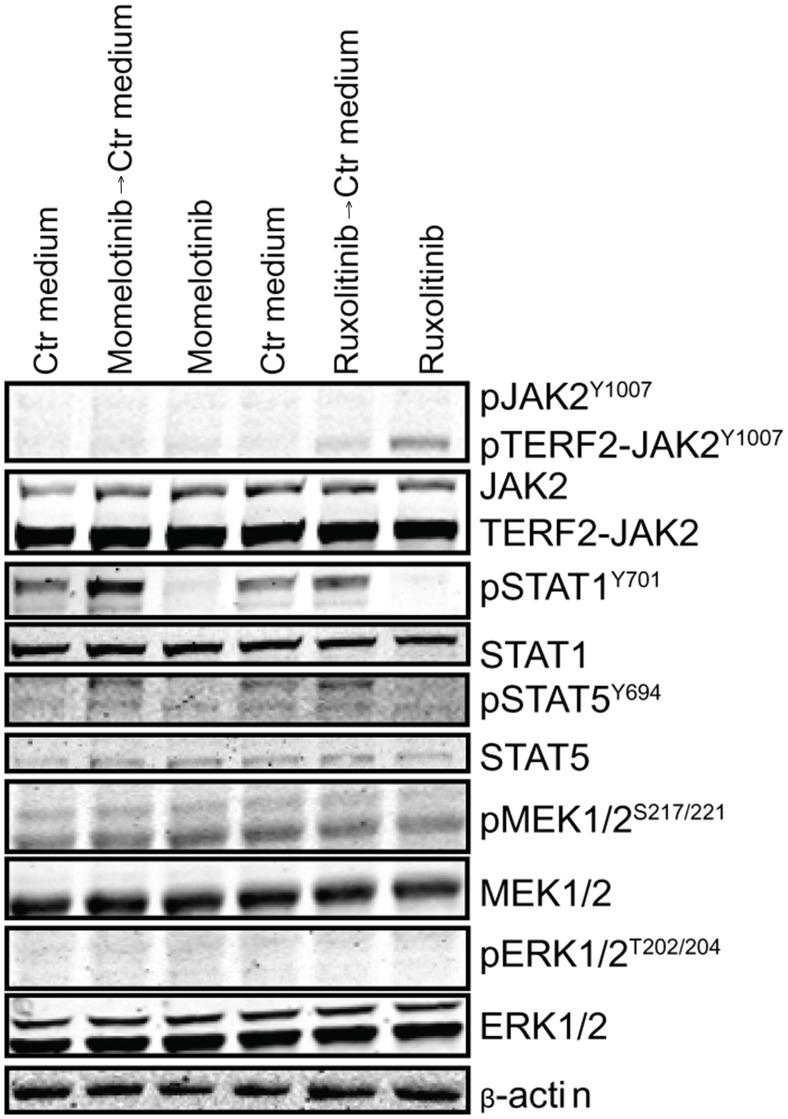
Accumulation of pJAK2^Y1007^ results in a rebound effect of JAK2 *TERF2-JAK2* PDX cells were incubated for four hours with or without 1.5 μM momelotinib or 0.75 μM ruxolitinib, after which cells were washed to remove the JAK inhibitors. Half of the cells were exposed for another 1.5 hours to 1.5 μM momelotinib or 0.75 μM ruxolitinib, whereas the other cells were incubated in vehicle control (Ctr) medium. Protein expression levels were examined by western blot (25 μg lysate).

Exposure of primary leukemic cells, harboring *TERF2-JAK2* or *PAX5-JAK2* translocations, to momelotinib and ruxolitinib resulted in accumulation of phosphorylated JAK2^Y1007^ fusion proteins (Figure 2C and 2D). Wash out of both inhibitors induced a slight rebound effect with upregulation of pSTAT1^Y701^ and pSTAT5^Y694^ in *TERF2-JAK2* cells (Figure 4A, Supplementary Figure 10). This rebound effect was also observed in the *JAK2^V617F^*-positive leukemic cell line HEL (Supplementary Figures 11 and 12). Phosphorylated JAK2^Y1007^ accumulated upon exposure to ruxolitinib. Removal of the inhibitor resulted in reactivation of JAK2 signaling, observed by a clear increase in phosphorylated STAT5^Y694^ levels within 4 hours (time point 100 hours; Supplementary Figure 11A and 11B). The inhibitory effect of momelotinib was more transient compared to ruxolitinib, resulting in an earlier reactivation of JAK2, observed by high levels of phosphorylated STAT5^Y694^ after 48 hours of momelotinib exposure (Supplementary Figure 11A).

### Mesenchymal stromal cells protect against JAK inhibitors

**Figure 5:**
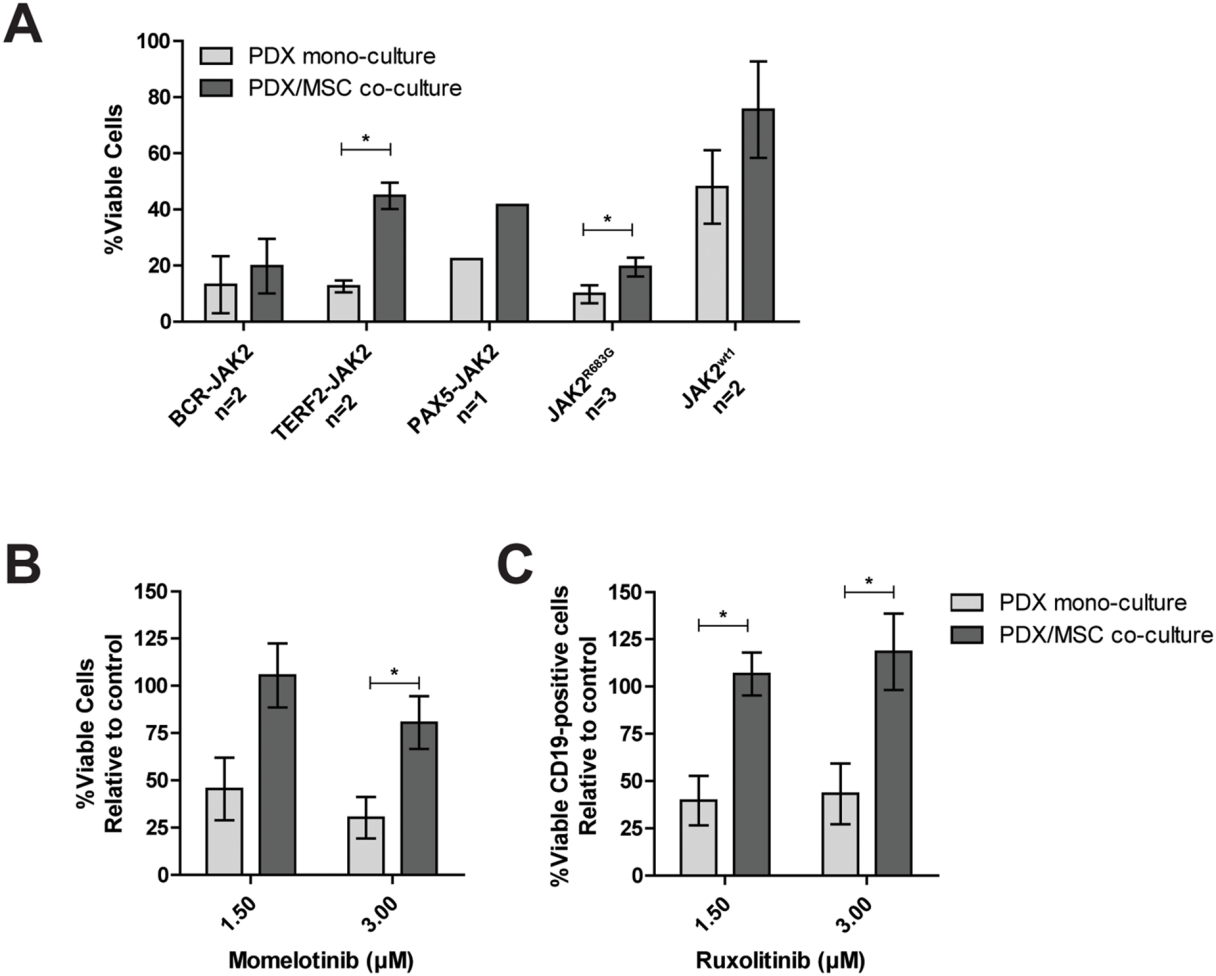
Efficacy of JAK inhibitors in co-culture The response of CD19^+^ PDX cells, co-cultured with MSCs (hCD19^-^), to increasing concentrations of momelotinib and ruxolitinib, was assessed after four days of culture using flow cytometry. Cells were stained with Brilliant Violet 421 anti-human CD19 antibody, FITC Annexin V, and PI. Viability was calculated relative to vehicle controls. **(A)** Survival of PDX cells in mono-culture, or PDX/MSC co-culture. Individual samples were tested in duplicate. Bars represent the mean±SEM of two *BCR-JAK2*, two *TERF2-JAK2*, one *PAX5-JAK2*, three *JAK2^R683G^* and two *JAK2^wt^* samples. Cell viability of samples in mono-culture versus co-culture was compared using the independent sample T-test. ^*^p≤0.05. **(B-C)** The effect of 1.5 μM and 3.0 μM momelotinib or ruxolitinib on the viability of PDX cells with *JAK2* translocations in mono-culture, or in co-culture with primary MSCs. Mean±SEM of three *JAK2* translocated samples is shown. Cell viability of samples was compared using independent sample T-test. ^*^p≤0.05.

Leukemic cells reside in the bone marrow microenvironment, where they disrupt normal hematopoietic stem cell niches [27]. This abnormal niche protects ALL cells against chemotherapy [28, 29]. To study whether the bone marrow microenvironment protects against JAK inhibitors, we mimicked this niche by co-culturing PDX cells with bone marrow mesenchymal stromal cells (MSCs) derived from a leukemia patient. Survival of leukemic cells was improved in co-cultures together with MSCs compared to leukemic cells cultured without MSCs (Figure 5A). In these PDX/MSC co-cultures, JAK inhibitors decreased leukemic cell survival (Supplementary Figure 13A-13G). However, leukemic cells were more resistant to ruxolitinib in PDX/MSC co-culture compared to culture without MSCs (P<0.05). A similar trend was observed for momelotinib (Figure 5B and 5C, Supplementary Figure 13H and 13I).

### Different outgrowth pattern in xenografts

**Figure 6:**
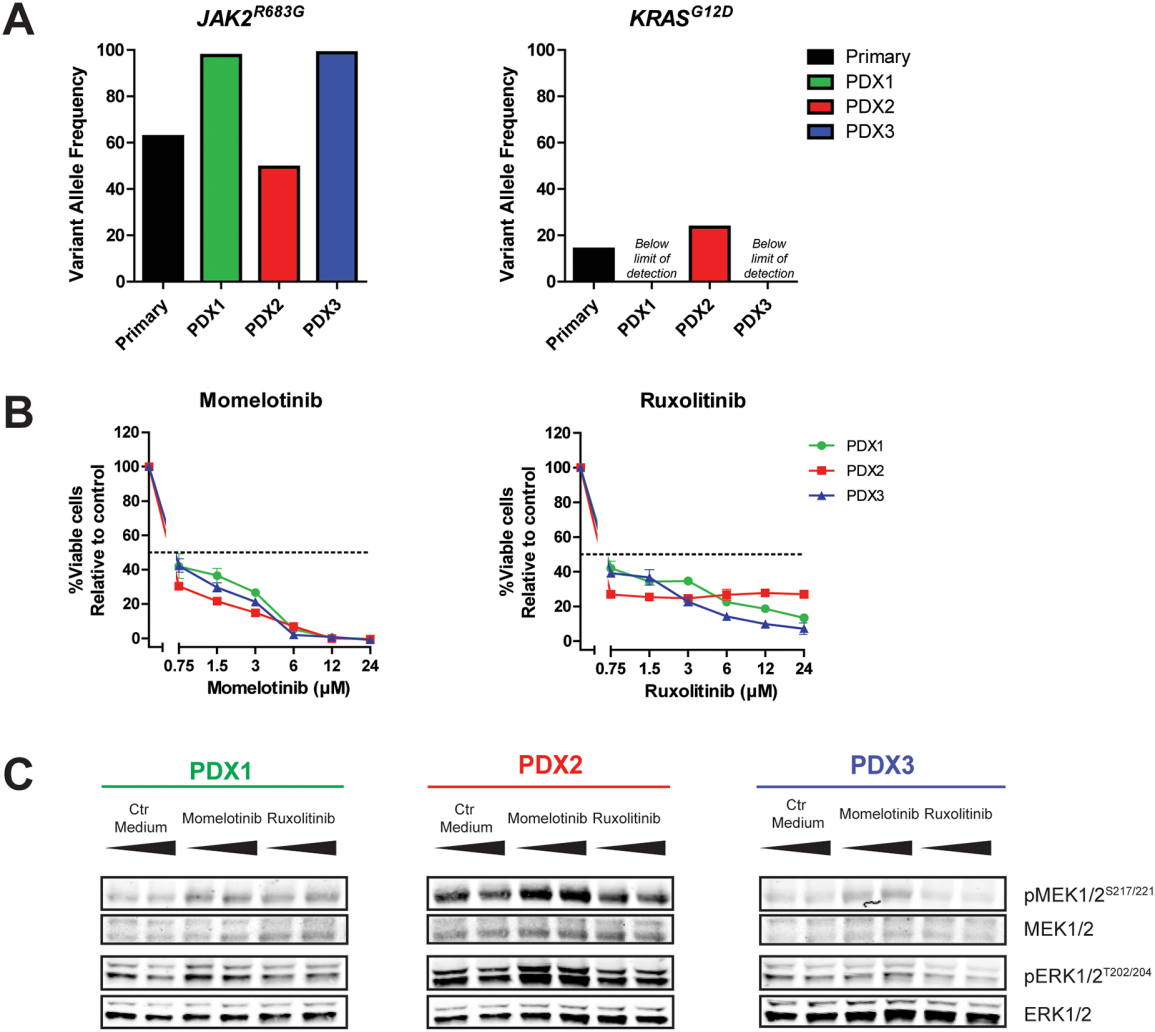
Outgrowth pattern of JAK2 mutated samples in xenografts **(A)** VAF of *JAK2^R683G^* and *KRAS^G12D^* in primary leukemic cells and three PDX samples of the same patient was determined using targeted amplicon sequencing. **(B)** PDX cells were pre-incubated for 1 hour with 25 ng/ml TSLP, after which they were exposed for four days to indicated concentrations of momelotinib or ruxolitinib in the presence of TSLP. Cell viability was measured using an MTT assay. Cell survival was calculated relative to vehicle treated controls. Samples were tested in duplicate. Mean±SD of each sample is shown. **(C)** Protein expression levels of PDX samples, exposed to 1.5 μM momelotinib or 0.75 μM ruxolitinib for four hours.

The outgrowth patterns of primary leukemic cells (>90% blast purity) in three NSG mice per patient was determined by paired-end deep-sequencing of *JAK2* hot spot regions (exon 16, 20, 21 and 23; median read depth 554, 465, 411 and 593, respectively). PDX cells originating from a *JAK2^R863G^* mutated case had a different VAF profile compared to the original patient sample (Figure 6A, Supplementary Figure 14A). The primary sample contained a major *JAK2^R683G^* clone at a VAF of 63% and a minor *KRAS^G12D^* clone at VAF 14%. In two out of three PDX samples generated, the VAF of the *JAK2^R683G^* mutation increased to 98% (PDX1) and 99% (PDX3), whereas in the remaining PDX sample the VAF decreased to 49% (PDX2). In contrast, the *KRAS^G12D^* mutation increased to a VAF 23% in this PDX2 sample, whereas this mutation was not detected in PDX1 and PDX3. The reduced VAF of the *JAK2* clone in PDX2 did not result in a decreased efficacy of momelotinib or ruxolitinib (Figure 6B). However, levels of pMEK1/2^S217/221^ and pERK1/2^T202/204^ in this sample were increased compared to the other two PDX samples (Figure 6C). Exposure to both JAK inhibitors did not decrease the levels of phosphorylated MEK and ERK.

Sanger sequencing of *JAK2^R683S^* and *JAK2^R683T^* PDX models also indicated a change in the VAF of PDX cells compared to the primary sample. In PDX cells from three NSG mice injected with *JAK2^R683S^* cells, the A/T peak ratio at nucleotide position 2049 differed, suggesting heterogeneity in frequency of *JAK2* mutations between samples (Supplementary Figure 14B). Strikingly, two mice injected with *JAK2^R683T^* mutated leukemic cells developed *JAK2* wildtype leukemia (Supplementary Figure 14C). Although the *JAK2* mutation was lost, *CRLF2* expression levels remained high (Supplementary Figure 14D and 14E). TSLP stimulation activated the JAK2 pathway signaling (Supplementary Figure 14F), but cells were not sensitive for JAK2 inhibition (Supplementary Figure 14G and 14H).

## DISCUSSION

This study aimed to evaluate the clinical need (frequency of lesions and prognostic value) and potential of JAK inhibitors in pediatric BCP-ALL. For this purpose pediatric BCP-ALL patients were screened for *JAK2* lesions. *JAK2* point mutations were found in 3.6% of our BCP-ALL patients, of which the majority were *JAK2^R683^* mutations. These mutations were solely detected in *BCR-ABL1*-like, B-other and high hyperdiploid patients, but not in *KMT2A-AFF1*, *BCR-ABL1*, *ETV6-RUNX1* or *TCF3-PBX1* patients. *JAK2* translocations were detected in the poor prognostic *BCR-ABL1*-like group, but not in non-*BCR-ABL1-like* B-other cases. The prognosis of patients with *JAK2* aberrations was as poor as *JAK2* wildtype *BCR-ABL1*-like and non-*BCR-ABL1*-like B-other patients.

*JAK2* mutations were not detected as frequently in our DCOG/COALL sequencing cohort as reported for COG high-risk cohorts [5, 10]. Two independent classifiers are used to describe *BCR-ABL1*-like BCP-ALL [30–32]. Differences in genetic ancestry between the American COG and European DCOG/COALL cohorts likely affected the signatures to classify patients as *BCR-ABL1-*like. Hence, genetic differences, or more specifically the lack of Hispanic/Latino cases [10, 33], might explain the lower frequencies of JAK2 mutations, as well as the difference in treatment outcome.

A targeted approach was used to detect *JAK2* translocations. All cases with high *JAK2* expression levels harbored one of the known fusion genes, making it therefore unlikely that novel fusion genes were missed.

The poor outcome of cases harboring *JAK2* lesions underline the clinical relevance of activated *JAK2*. The therapeutic potential of JAK2 was demonstrated by efficacy of JAK inhibitors in *JAK2* mutated and *JAK2* translocated primary BCP-ALL cells. *JAK2* translocated cells were sensitive to both JAK inhibitors, independent of cytokine activation. In contrast, the efficacy of JAK inhibitors in *JAK2* mutated cells highly depended on CRLF2 activation by TSLP, which is in concordance with previous reports [3, 5, 7, 8, 18]. These results highlight the importance of TSLP in *in vitro* and *in vivo* studies involving *JAK2* mutations. Since human CRLF2 cannot be activated by mouse TSLP, conventional mouse xenograft models are not suitable to test the efficacy of ruxolitinib and other JAK inhibitors on *JAK2* mutated cells. Absence of human TSLP in ALL xenograft mouse models might therefore explain the disappointing efficacy of JAK inhibitors in *in vivo* models [19–22]. The recently engineered xenograft model which synthesizes human TSLP may overcome this limitation [34].

Despite the need for human TSLP, we and others observed engraftment of primary *JAK2* mutated cells in NSG mice, giving rise to leukemia within months after intra-femoral injection [19, 20, 22]. This implies that the proliferation of *JAK2* mutated leukemic cells in the mouse bone marrow does not (solely) depend on CRLF2 signaling, but may be supported by activation of alternative pathways [35, 36]. This argues against targeted monotherapy, as cells might escape via an alternative pathway, such as the RAS pathway [36, 37]. This is further strengthened by a study in Down syndrome ALL, in which a switch was observed from a *JAK2* mutation at initial diagnosis to a *RAS*-mutation at relapse [38]. In the current study, we also observed co-occurrence of *JAK2* and *KRAS* lesions in non-Down syndrome BCP-ALL cells. More importantly, these *JAK2* and *KRAS* mutations showed different outgrowth patterns in NSG xenograft models. Co-occurrence of these mutations suggests that a combination of JAK and RAS pathway inhibitors may be more effective for *JAK2*-aberrant cases. Moreover our results indicate that *JAK2* mutations by itself might not be drivers of leukemic outgrowth, which is also corroborated by the outgrowth of *JAK2* wildtype leukemic cells in mice injected with *JAK2^R683T^* cells. Taken together, these data imply that 1) the expansion properties of *JAK2* mutated leukemic cells vary, 2) other subclones (e.g. *KRAS^G12D^*) can grow out at the expense of the major *JAK2* mutated clone, 3) *JAK2* mutations can get lost while leukemia progresses. Therefore, *JAK2* mutations are most likely secondary lesions, in contrast to *JAK2* translocation, which are primary lesions. The selection of alternative pathways indicates a potential risk for use of JAK inhibitors in *JAK2* mutated cases without detailed monitoring of the mutational status of leukemic cells in time.

Interestingly, we observed a difference in the cytotoxicity of momelotinib and ruxolitinib. Both agents fully inhibited JAK/STAT signaling by downregulation of phosphorylation levels of STAT1 and STAT5. However, only momelotinib affected *JAK2* wildtype and non-TSLP stimulated *JAK2* mutated cells, regardless of activation of STAT5. Therefore, it is likely that momelotinib also affects alternative targets [39, 40]. In addition, our data revealed that exposure to JAK inhibitors, especially ruxolitinib, resulted into accumulation of phosphorylated JAK2^Y1007^, which consequently induced a profound re-activation of downstream STAT signaling. Accumulation of phosphorylated JAK2^Y1007^ is in concordance with other reports [21, 41–43]. The rebound effect observed in the present study identifies an important limitation of these agents. As type I inhibitors bind JAK2 in its active conformation, reactivation of JAK2 may be overcome by type II inhibitors, which bind JAK2 in its inactive conformation [18, 44]. Preclinical *in vitro* and *in vivo* data indicate high efficacy of the type II inhibitor CHZ868. Clinical data of this inhibitor and other novel inhibitors are warranted.

Besides studying the mechanism of action, we showed influences of the bone marrow microenvironment on the efficacy of JAK inhibitors. Microenvironment-induced resistance of leukemic cells has been reported for conventional drugs, e.g. prednisolone and asparaginase, for imatinib in *BCR-ABL1*-positive cells, and for JAK inhibitors in the present study [45–49]. These data imply that the local tumor environment can stimulate a survival program, which may provide leukemic cells a way to escape from JAK inhibitors.

Taken together, *JAK2* lesions are infrequently found in children with newly diagnosed BCP-ALL and are mainly restricted to *BCR-ABL1*-like and non-*BCR-ABL1-like* B-other cases (negative for sentinel cytogenetic lesions). Although mutations and translocations represent biologically distinct entities, both may be targetable by JAK inhibitors. Indeed, we showed efficacy of momelotinib and ruxolitinib in both cell types. As both momelotinib and ruxolitinib have a short half-life (less than half a day), the observed rebound effect may be therapeutically of risk [50, 51]. Although it should be confirmed by functional *in vivo* studies, our data suggest that the effect of JAK inhibition may be compromised by microenvironment-induced resistance and induction of alternative survival pathways. In conclusion, JAK inhibitors may be considered to be added to, but not substitute, chemotherapy for patients with *JAK2* aberrations, especially for those at high risk of relapse due to high MRD levels upon induction therapy. Therapeutic monitoring for activation of alternative pathways (e.g. RAS) is highly recommended.

## MATERIALS AND METHODS

### Primary patient-derived material

Bone marrow and/or peripheral blood samples were obtained from children (1-18 years) with newly diagnosed ALL. Written informed consent was obtained from parents or guardians to use excess diagnostic material for research purposes, as approved by the institutional review board. These studies were conducted in accordance with the Declaration of Helsinki. Mononuclear cells were isolated and processed as described previously [52]. Samples were enriched towards > 90% leukemic cells by depletion of non-leukemic cells using immunomagnetic beads. The major cytogenetic subtypes were determined using fluorescent *in situ* hybridization, (RT-) PCR, and the 110-probeset gene expression classifier [31]. Patients were treated according to the DCOG ALL8, ALL9 or ALL10 protocol, or the COALL-06-97 and COALL-07-03 study protocols [53–57]. Patient characteristics were provided by the central study centers of DCOG (The Hague, the Netherlands) and COALL (Hamburg, Germany).

Mesenchymal stromal cells (MSCs) were isolated from the bone marrow aspirate of a *BCR-ABL1*-like patient as described previously [58]. Purity of MSCs was assessed by negativity for the hematopoietic markers CD34, CD45, and CD19, and positivity for the mesenchymal markers CD13, CD29, CD44, CD54, CD73, CD90, CD105, CD146 and CD166, as detected by flow cytometry (MACS Quant). Expression was measured using the human mesenchymal stem cell marker antibody panel (R&D Systems, Minneapolis, Minnesota, USA), and CD13-APC, CD29-APC, CD34-PE, CD54-PE, CD73-PE, IgG1-PE, and IgG1-APC (BD Biosciences, San Jose, California, USA).

### Cell lines

The human erythroleukemia cell line HEL was obtained from the German Collection of Microorganisms and Cell lines (DSMZ, Braunschweig, Germany) and cultured in RPMI-1640 medium, supplemented with 10% fetal calf serum (Bodinco BV, Alkmaar, Netherlands), 100 units/ml penicillin, 100 μg/ml streptomycin and 0.125 μg/mL fungizone (Life Technologies, Bleiswijk, Netherlands). The identity of the cell line was routinely verified by DNA fingerprinting. Additionally, presence of *Mycoplasma* was excluded every 25 passages by PCR.

### Patient-derived-xenograft cells

Primary leukemic cells were transplanted by intra-femoral injection in 7-12 weeks old female NOD.Cg-Prkdc^scid^Il2rg^tm1Wjl^/SzJ (NSG) mice (Charles River, Wilmington, Massachusetts, USA; three NSG mice per patient), as approved by the animal ethics committee (EMC 2863). Mice were sacrificed upon overt leukemia or 6 months after injection. Leukemic cells were isolated from bone marrow and spleen. The percentage of human leukemic cells was analyzed by flow cytometry (APC anti-human CD19, PE anti-human CD45 and FITC anti-mouse CD45; Biolegend, London, UK) and May-Grünwald-Giemsa staining. Patient-derived-xenograft cells (PDX) were enriched for > 90% human leukemic cells using anti-human-CD19 immunomagnetic beads (MACS Miltenyi, Leiden, Netherlands).

### JAK2 mutations

*JAK2* mutations status was analyzed in 461 BCP-ALL cases, representing the major cytogenetic subtypes (6% *BCR-ABL1*, 17% *BCR-ABL1*-like, 15% non-*BCR-ABL1*-like B-other, 27% *ETV6-RUNX1*, 27% high hyperdiploid, 3% *KMT2A-AFF1*, 6% *TCF3-PBX1*). Genomic DNA was isolated using Trizol reagent (Life Technologies), or in some cases using the DNeasy blood and tissue kit (Qiagen) according to manufacturer's instructions, and quantified using the Qubit dsDNA Broad Range Assay Kit (Life Technologies). 100-250 ng genomic DNA was used to prepare sequencing libraries according to manufacturer's instructions. Successful library preparation, correct amplicon length and concentration were assessed using the Labchip GX genomic analyzer (Caliper Life Sciences Benelux N.V.) with the HT DNA 12K Reagent Kit, Version 2. Samples were pooled equi-molarly and sequenced on an Illumina MiSeq in paired-end reads of 250bp each. The custom amplicons covered the exons 16, 20, 21 and 23 and represent the mutational hotspot region of *JAK2*.

Sequence reads were exported in fastq format and, aligned to the standard 1000 genomes human reference sequences (version b37, from the GATK resource bundle provided by the Broad institute, USA), using BWA version 0.7.10 and the GATK indel realigner version 3.3-0. Freebayes version 0.9.18-24, Varscan version 2.3.7, Bcftools version 1.0, and GATK version 3.3-0 were used to call single nucleotide variants. The resulting variant call format files were annotated using snpEff and snpSift version 4.1a and the dbNSFP version 2.7 database. Variants were combined and filtered based on several criteria using an in-house developed pipeline: variants were excluded if they were not located in targeted regions, were reported by only one caller, had coverage of < 100 reads, or had < 10 reads supporting the variant allele. Furthermore, variants had to occur at least once with a VAF above 2% in any sample and to be distributed equally between runs according to a chi-square test. Additionally only variants were taken into account if they were reported in the COSMIC V73 GRCh37 database, lead to an amino acid change, were unlikely to be a germline variant and not a known SNP. SNPs were defined as having a mean population frequency of ≥ 5% across the 1000Gp1 complete human population, 1000Gp1 population of European decent, and the ESP-6500 population of European decent allele frequency databases. Variants found in ≥ 10 samples with a mean and median VAF > 40% were labelled as possible germline. Sufficient coverage was crucial to detect subclonal mutations, which could be reliably detected ≥ 5% VAF in on average 91% of the cases. Detection of variants with a frequency of 1% was limited to a smaller group (26%), and is therefore a conservative estimate in the present study.

Genomic DNA of PDX cells was used to identify *JAK2* mutations in exon 16. Samples were analyzed by Baseclear B.V. using Sanger sequencing (forward primer 5’-ATGCCTCCAAATTATTATACTATCA-3’, reverse primer 5’-ATCACCTCACAGTCCATGGTTAT-3’).

### JAK2 translocations

Presence of ten recurrently reported *JAK2* translocations was examined by RT-PCR in 153 BCP-ALL cases, negative for sentinel BCP-ALL associated lesions (n=77 *BCR-ABL1*-like, n=76 non-*BCR-ABL1*-like B-other). Total RNA was extracted from leukemic cells using Trizol reagents (Life Technologies). cDNA was synthesized using random hexamers and oligodT primers, and M-MLV reverse transcriptase (Promega, Leiden, Netherlands). RT-PCR was performed in a final volume of 25 μl, containing 0.3 μM forward primer, 0.3 μM reverse primer, 200 μM dNTPs, 1x PCR buffer II, 4 mM MgCl_2_, 0.125 μl AmpliTaq DNA polymerase (Promega) and 2.5 μl cDNA. Primer sequences are shown in Supplementary Table 1. PCR products were Sanger sequenced by Baseclear B.V. (Leiden, Netherlands).

### CRLF2 status

*CRLF2* expression levels were determined by Affymetrix gene expression microarrays (U133 Plus 2.0; Santa Clara, California, USA), of a previously published cohort of pediatric BCP-ALL patients at initial diagnosis [59]. High *CRLF2* expression levels are indicative *P2RY8-CRLF2* and *IGH-CRLF2* translocations in different studies, though not all cases with high *CRLF2* mRNA expression levels harbor *CRLF2* rearrangements [10, 16, 59–61]. Of 406 cases, both *CRLF2* gene expression data and *JAK2* mutation status was available. Signal intensity of probeset 208303_s_at above the 90^th^ percentile levels was classified as *CRLF2* high, as described previously [59].

In addition, *CRLF2* expression in primary leukemic and PDX samples was determined using RT-qPCR and SYBR green. *CRLF2* expression (forward primer 5’-ACGGGGATCTCCTCTATG-3’, reverse primer: 5’-GAGGCGTTGG TGTCTCT-3’) was calculated relative to *RSP20* expression (forward primer 5’-AAGGGCTGAGGATTTT TG-3’, reverse primer 5’-CGTTGCGGCTTGTTAG-3’), using the comparative cycle time (Ct) method; 2^-DCt^ x 100%, whereby DCt = Ct_*CRLF2*_ – CT_*RPS20*_. RT-qPCR expression values were correlated to Affymetrix microarray data of probeset 208303_s_at.

### JAK2 expression levels

*JAK2* expression levels were analyzed using Affymetrix gene expression microarrays, in a previously published pediatric BCP-ALL cohort [59]. Signal intensity of probeset 205841_at was used to quantify *JAK2* gene expression.

### *Ex vivo* drug cytotoxicity assays

Cell-intrinsic resistance towards momelotinib and ruxolitinib (Selleck Chemicals, Kirby Drive, Houston, USA) was evaluated as described previously [52]. Briefly, leukemic cells were exposed to a concentration range (24μM to 750 nM) of these compounds for four days and cytotoxicity was quantified using 3-(4,5-dimethylthiazol-2-yl)-2,5-diphenyltetrazolium bromide (MTT). For TSLP stimulation, cells were pre-incubated for 1 hour with 25 ng/ml TSLP (R&D systems, Oxon, UK). In addition to single cell cultures, leukemic cells (1^*^10^6^ cells) were co-cultured with primary MSCs (5^*^10^4^ cells) for four days in a 24 well plate in the presence of a dilution series of momelotinib and ruxolitinib. Cell survival was quantified using flow cytometry (MACSQuant, FlowJo 10.0.8r1), and cells were stained with Brilliant Violet 421 anti-human CD19 antibody (Biolegend), FITC Annexin V (Biolegend), and Propidium Iodide (PI; Invitrogen, Bleiswijk, Netherlands), as described previously [45].

### Western blotting

Leukemic cells were lysed in lysis buffer supplemented with freshly added protease and phosphatase inhibitors. 25μg (BCA method; Thermo Scientific) lysate was loaded on 10% mini protean precast gels (BioRad, Veenendaal, Netherlands), and transferred to a nitrocellulose membrane (Biorad). Primary antibody incubation was performed according to manufacturer's protocol. Anti-JAK2 (#3230), anti-phospho-JAK2-Tyr1007 (#4406), anti-phospho-STAT5-Tyr694 (#9351), anti-phospho-STAT1-Tyr701 (#9167), anti-Stat1 (#9175), anti-phospho-MEK1/2-Ser217/221 (#9154), anti-MEK1/2 (#4694), anti-phospho-Erk1/2-T202/204 (#4370), anti-Erk1/2 (#91078), and anti-αTubulin (#2144) were obtained from Cell Signaling Technology (Danvers, Massachusetts, USA). Anti-β-actin (ab6276) was obtained from Abcam (Cambridge, UK), and anti-STAT5 (sc-835) from Santa Cruz (Heidelberg, Germany). Blots were stained with secondary antibodies (IRDye 680RD- or 800CW-labelled anti-rabbit and IRDye 680RD- or 800CW-labelled anti-mouse; Li-Cor Biosciences, Leusden, Netherlands) and scanned using the Odyssey infrared imaging system (Li-Cor Biosciences). To reprobe membranes, they were stripped in NewBlot Nitrocellulose stripping buffer (Li-Cor Biosciences) according to manufacturer's protocol. BCR-JAK2, PAX5-JAK2 and TERF2-JAK2 proteins were separated from wildtype JAK2 based on size (~94, 57, 95 and 125 kDa, respectively).

### Statistics

Cumulative incidence of relapse (CIR) was estimated using a competing risk model. Relapse and non-response (counted at day 79) were considered as event, and death as competing event. The Gray's test was applied to test for equality of CIRs [62]. Outcome analyses were performed in R 3.0.1, using the packages cmprsk version 2.2-7 [63], mstate version 0.2.7 [64], and survival version 2.37-4 [65].

## SUPPLEMENTARY MATERIALS FIGURES AND TABLES



## Abbreviations

BCP-ALL: B-cell precursor acute lymphoblastic leukemia
CIR: cumulative incidence of relapse
CRLF2: cytokine receptor-like factor 2
JAK2: Janus kinase 2
MSCs: mesenchymal stromal cells
MRD: minimal residual disease
MTT: 3-(4,5-dimethylthiazol-2-yl)-2,5-diphenyltetrazolium bromide
NSG: NOD.Cg-Prkdc^scid^Il2rg^tm1Wjl^/SzJ
PDX: patient-derived-xenograft
STATs: signal transducers of transcription
VAF: variant allele frequency
