# Supplementary material for: JAK2 aberrations in childhood B-cell precursor acute lymphoblastic leukemia
Source: Oncotarget. 2017 Sep 16;8(52):89923–38. doi: 10.18632/oncotarget.21027 (PMC5685720; doi:10.18632/oncotarget.21027)
Supplement: Supplementary file 1 [file oncotarget-08-89923-s001.pdf]

JAK2 aberrations in childhood B-cell precursor acute lymphoblastic leukemia

SUPPLEMENTARY MATERIALS

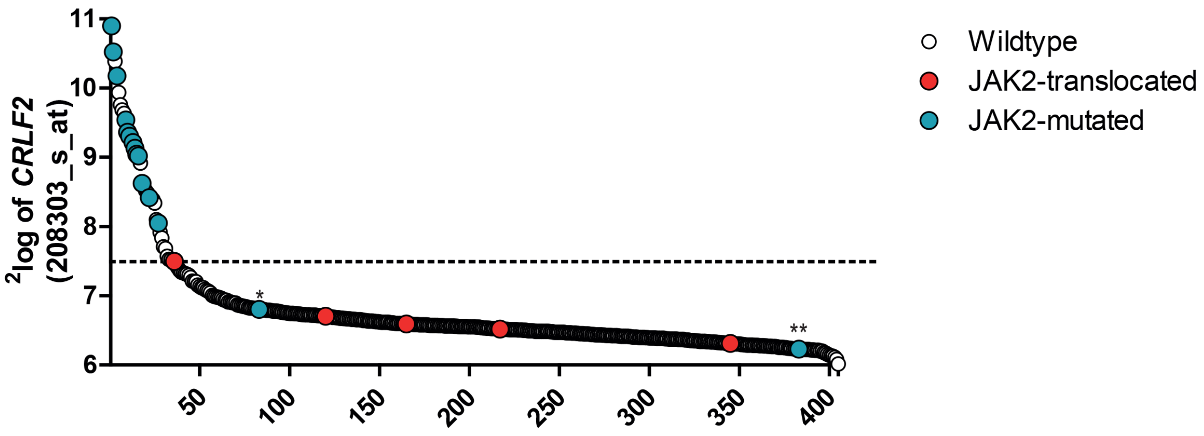

Supplementary Figure 1: CRLF2 expression values. ²log expression levels of Affymetrix probeset 205841_at in 405 pediatric BCP-ALL cases, which were tested for *JAK2* aberrations. * represents patient A159. ** represents patient A521.

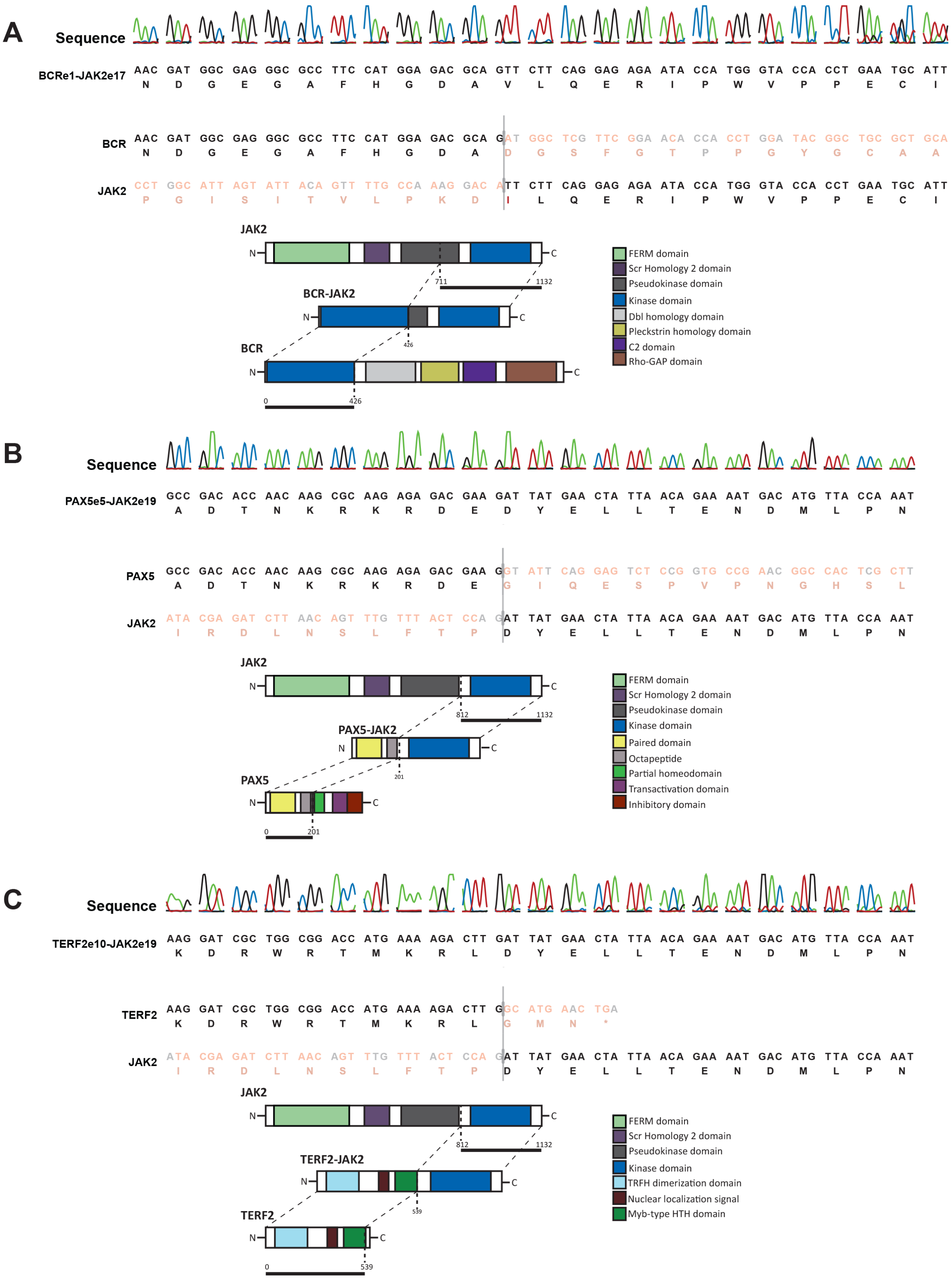

Supplementary Figure 2: Sequencing results of JAK2 translocations. **(A-C)** Presence of fusion genes was examined on cDNA level using RT-PCR, followed by Sanger sequencing to detect *BCR-JAK2*, *PAX5-JAK2* and *TERF2-JAK2* translocations.

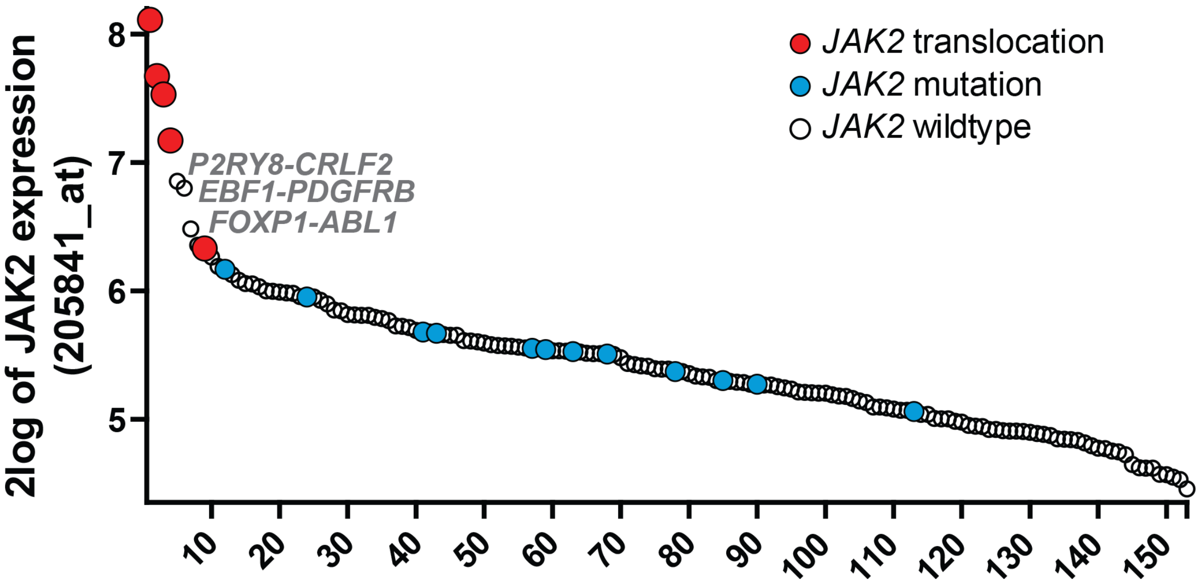

Supplementary Figure 3: JAK2 expression values. ^{2}log expression levels of Affymetrix probeset 205841_at in 77 *BCR-ABL1*-like and 76 B-other cases, which were tested for *JAK2* translocations. *JAK2* mutation status is also indicated.

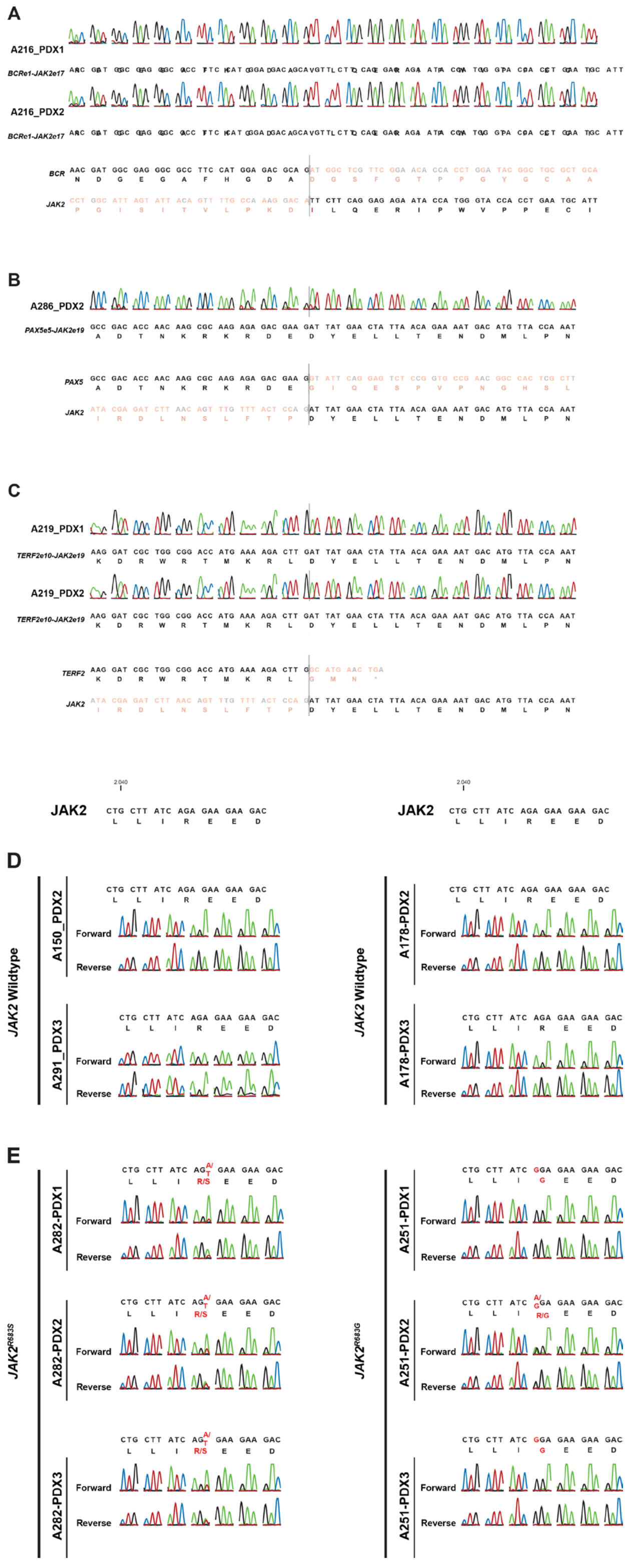

Supplementary Figure 4: JAK2 aberrations in PDX cells. **(A-C)** Presence of *JAK2* translocation in PDX cells was validated on cDNA level using Sanger sequencing of cDNA. **(D-E)** Genomic DNA of PDX cells was used to identify the absence (D) or presence (E) of *JAK2* mutations in exon 16.

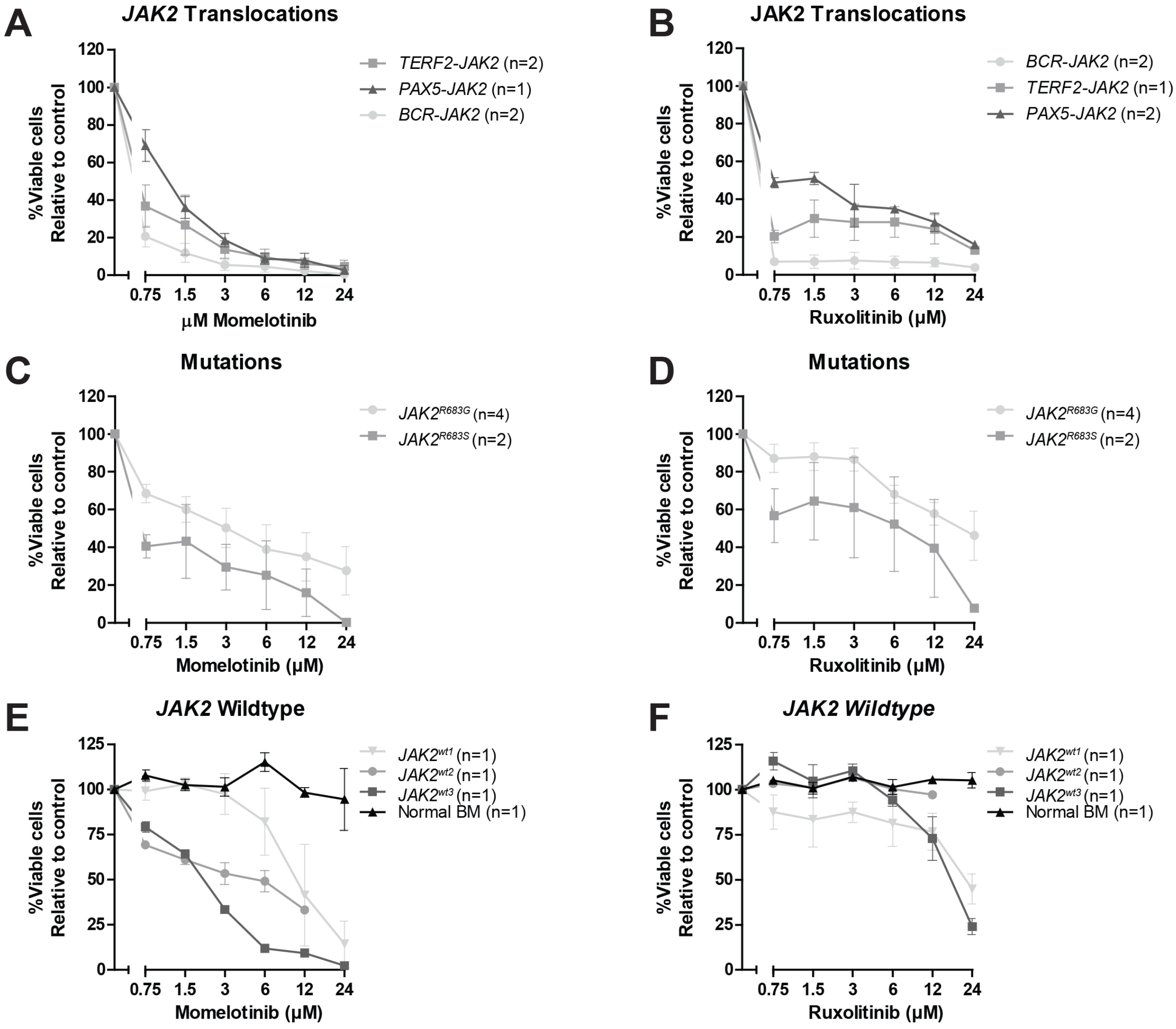

Supplementary Figure 5: The efficacy of JAK inhibitors on JAK2 translocated and mutated cells per type of aberration. Leukemic (PDX or primary patient) cells were incubated for four days to indicated concentrations of momelotinib or ruxolitinib, after which viability was measured using an MTT assay. Sensitivity of exposed cells was calculated relative to vehicle treated controls. Individual samples were tested in duplicate. Data derived from samples with identical *JAK2* lesions were grouped for analyses. **(A-B)** Efficacy of momelotinib and ruxolitinib on PDX cells with *JAK2* translocations: *BCR-JAK2* (n=2), *TERF2-JAK2* (n=2) and *PAX5-JAK2* (n=1). Mean±SD of individual samples (in duplicate) are shown. **(C-D)** Efficacy of momelotinib and ruxolitinib on *JAK2* mutated cells: *JAK2*^{R683G} (n=4) and JAK2^{R683S} (n=2) Mean±SEM of individual samples is depicted. **(E-F)** Efficacy of momelotinib and ruxolitinib on *JAK2* wildtype PDX cells and normal bone marrow cells. Mean±SD of individual samples (in duplicate) are shown.

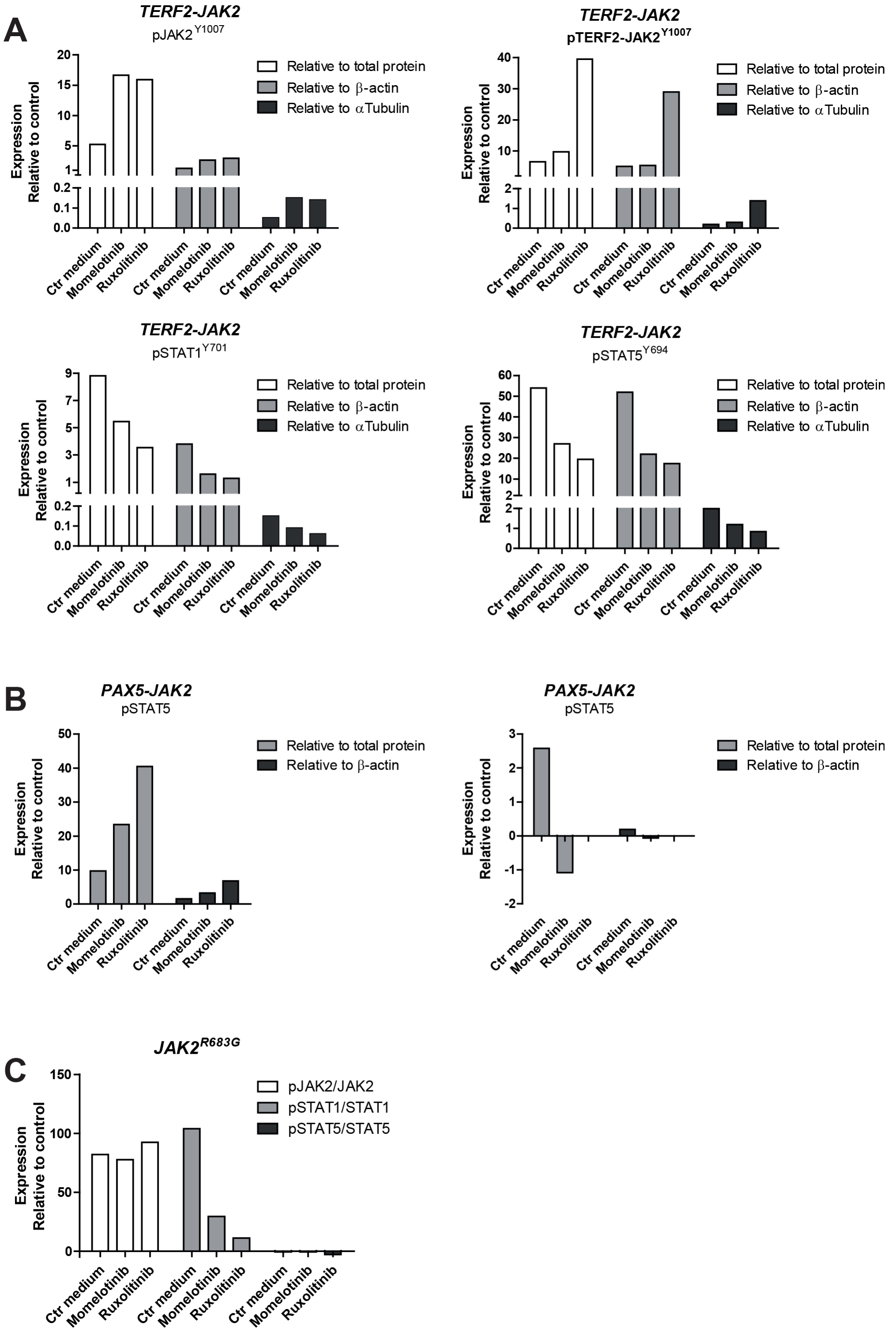

Supplementary Figure 6: Quantified expression levels western blots Figure 2. **(A-C)** *TERF2-JAK2, PAX5-JAK2* and *JAK2*^{R683G} PDX cells were exposed for four hours to vehicle control medium, 1.5 µM momelotinib or 0.75 µM ruxolitinib, after which (phosphorylated) TERF2-JAK2, PAX5-JAK2, JAK2, STAT1 and STAT5 levels were analyzed using western blot (25 µg lysate). Expression levels of phospho-proteins were quantified relative to total protein or loading control.

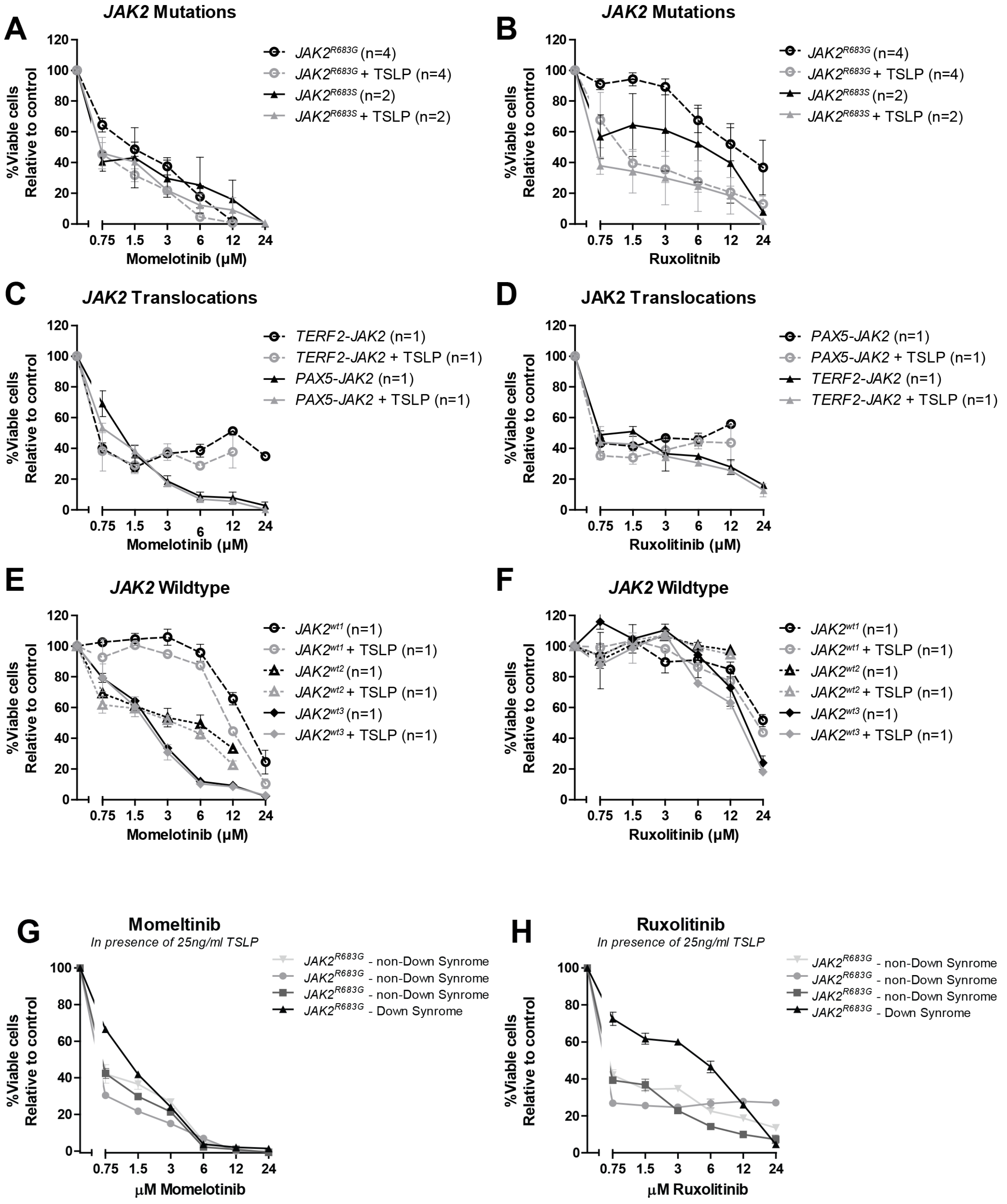

Supplementary Figure 7: The effect of TSLP stimulation on the efficacy of JAK inhibitors per type of aberration. Leukemic (PDX or primary patient) cells were pre-incubated for 1 hour without or with 25 ng/ml TSLP, after which they were exposed for four days to indicated concentrations of momelotinib or ruxolitinib. Viability was measured using an MTT assay. Sensitivity was calculated relative to vehicle treated controls. Individual samples were tested in duplicate. **(A-B)** Efficacy of momelotinib and ruxolitinib on *JAK2* mutated samples with or without TSLP pre-incubation: Mean±SEM four *JAK2*^{R683G} and two *JAK2*^{R683S} samples is shown. **(C-D)** Efficacy of momelotinib and ruxolitinib on cells with *JAK2* translocations (n=2). Mean±SD of sample duplicates from a *TERF2-JAK2* and *PAX5-JAK2* sample are shown. **(E-F)** Efficacy of momelotinib and ruxolitinib on *JAK2* wildtype PDX cells (n=3). Mean±SD of sample duplicates are shown.

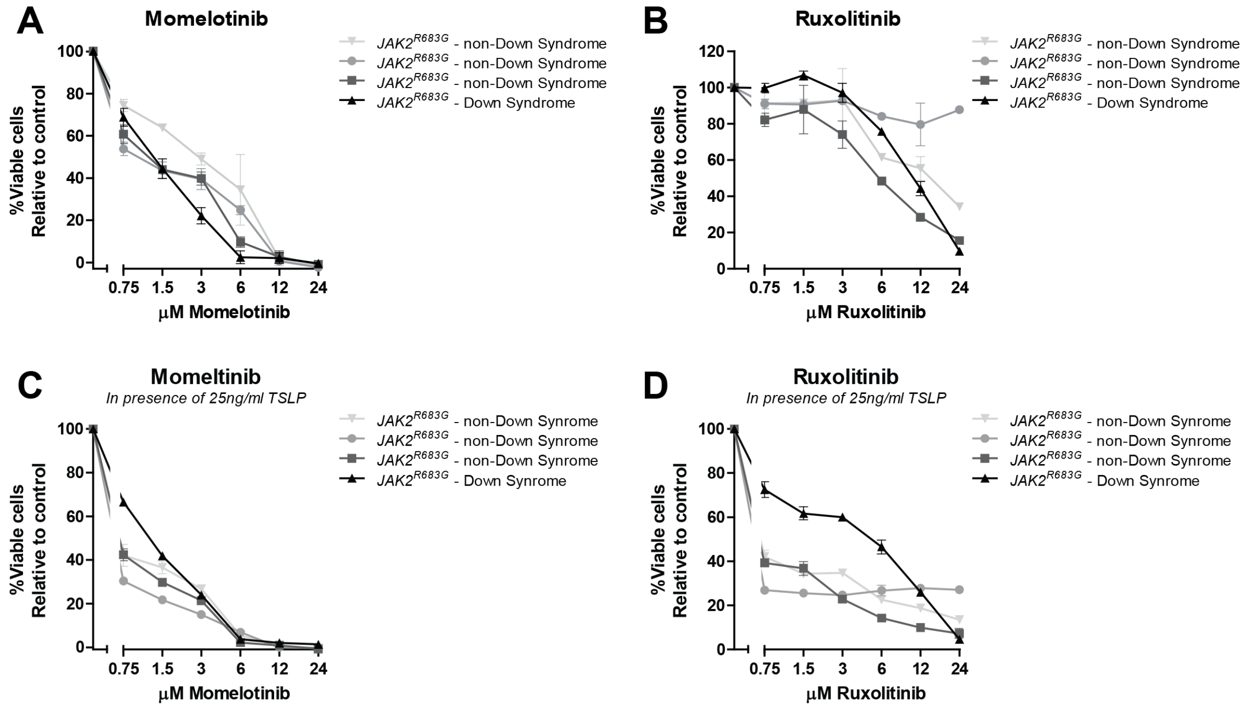

Supplementary Figure 8: Down syndrome and the efficacy of JAK inhibitors on JAK2 mutated cells. Cells were exposed for four days to indicated concentrations of momelotinib or ruxolitinib. Viability was measured using an MTT assay. Sensitivity of exposed cells was calculated relative to vehicle treated controls. Individual samples were tested in duplicate. Mean±SD of sample duplicates is shown.

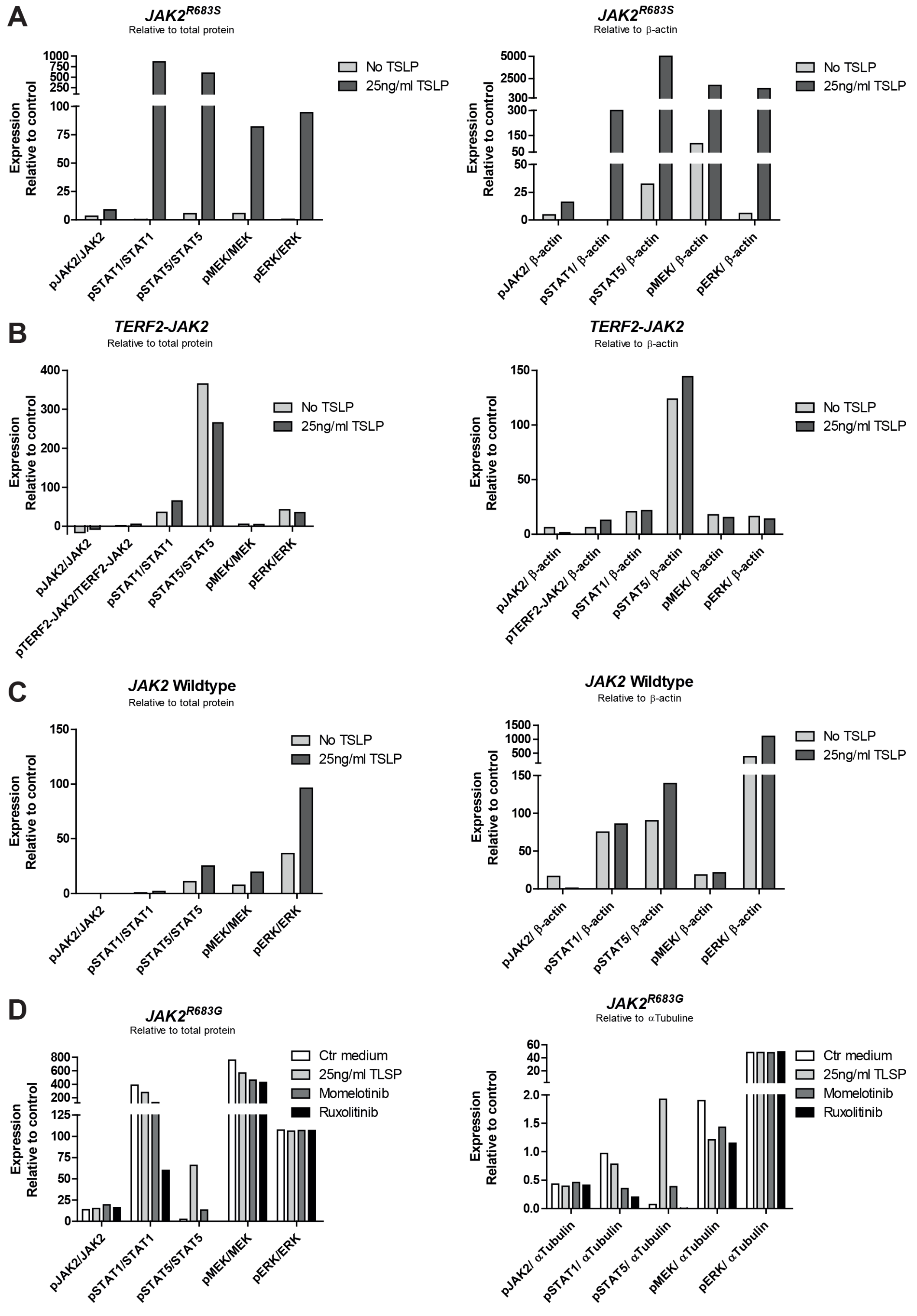

Supplementary Figure 9: Quantified expression levels western blots Figure 3. (A-C) Western blot of *JAK2*^{R683S}, *TERF2-JAK2* and *JAK2*^{wt} PDX cells with or without TSLP stimulation (25 ng/ml for 1 hour). Expression levels of phospho-proteins were quantified relative to total protein or loading control (β-actin). **(D)** *JAK2*^{R683G} cells were pre-incubated for 1 hour with or without 25 ng/ml TSLP, after which cells were exposed for four hours to vehicle control medium, 1.5 µM momelotinib or 0.75 µM ruxolitinib. Levels of (phosphorylated) JAK2, STAT1, STAT5, MEK1/2 and ERK1/2 were analyzed using western blot. Expression levels of phospho-proteins were quantified relative to total protein or loading control (α-Tubulin).

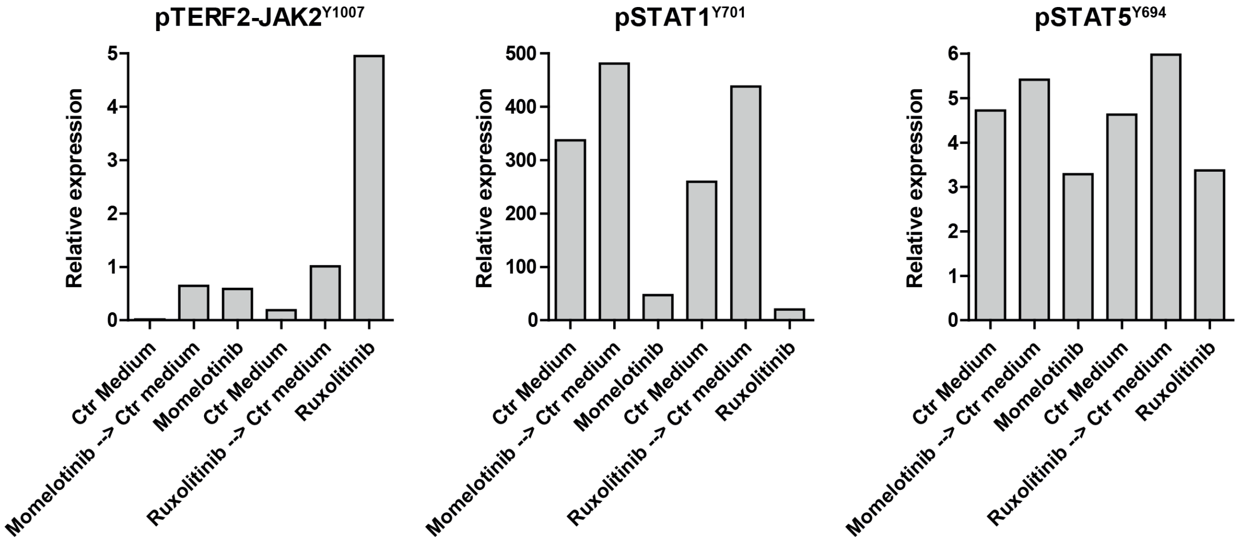

Supplementary Figure 10: Quantified expression levels western blots Figure 4. *TERF2-JAK2* PDX cells were incubated for four hours with or without 1.5 µM momelotinib or 0.75 µM ruxolitinib, after which cells were washed to remove the JAK inhibitors. Half of the cells were exposed for another 1.5 hours to 1.5 µM momelotinib or 0.75 µM ruxolitinib, whereas the other cells were incubated in vehicle control (Ctr) medium. Protein expression levels were examined by western blot (25 µg lysate). Expression levels of phosphoproteins were quantified relative to total protein.

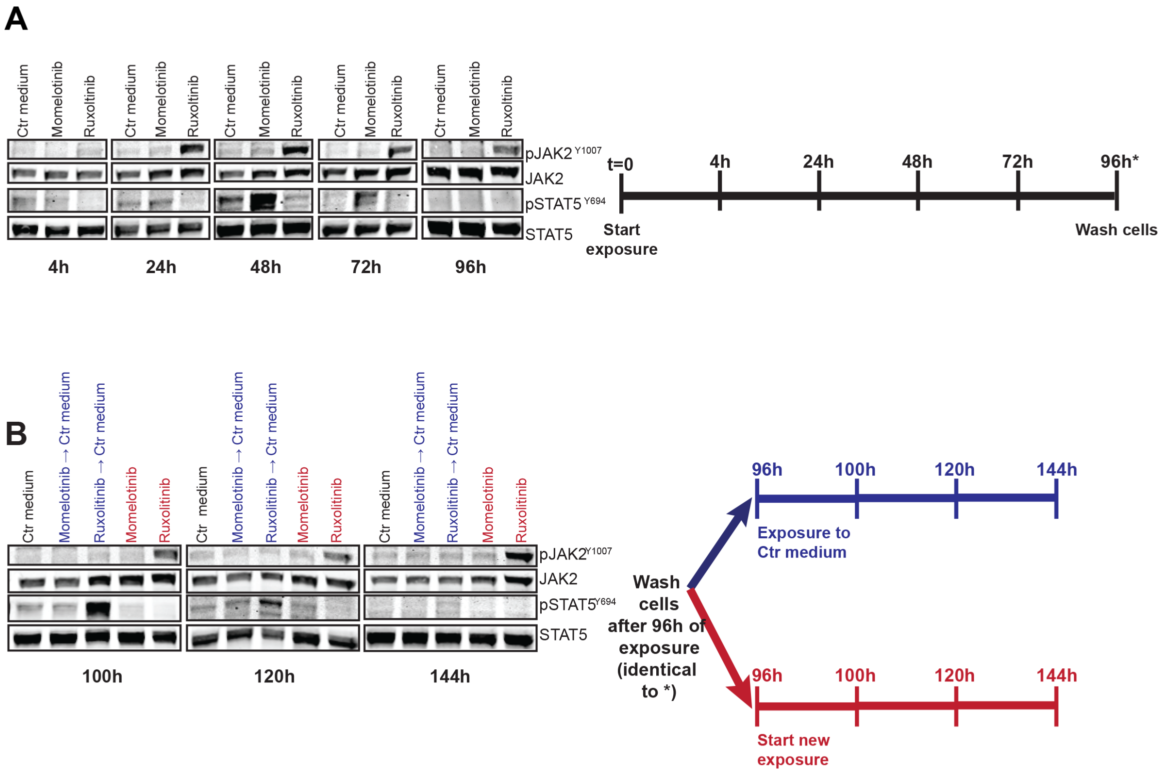

Supplementary Figure 11: Accumulation of pJAK2^{Y1007} results in a rebound effect of JAK2. (A) HEL cells were incubated with or without 1.5 µM momelotinib or 0.75 µM ruxolitinib for 4 hours, 24 hours, 48 hours, 72 hours and 96 hours, after which cells were lysed and protein expression levels were examined using western blot (25 µg lysate). **(B)** After 96 hours of exposure to JAK inhibitors (indicated by * in panel A), cells were washed in normal culture medium, to remove momelotinib and ruxolitinib. Subsequently, half of the cells were incubated with 1.5 µM momelotinib or 0.75 µM ruxolitinib for another 4 hours, or 24 hours, whereas the remaining cells were incubated in vehicle control (Ctr) medium. Protein expression levels were examined using western blot (25 µg lysate).

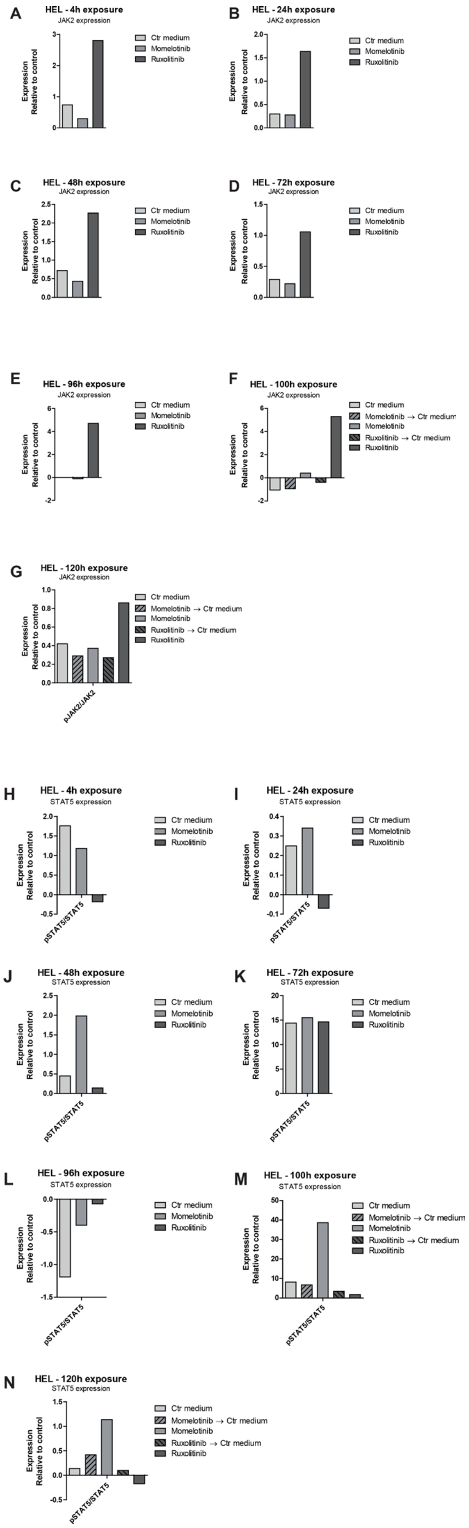

Supplementary Figure 12: Quantified expression levels western blots Supplementary Figure 8. (A-N) HEL cells were incubated with or without 1.5 µM momelotinib or 0.75 µM ruxolitinib for 4 hours, 24 hours, 48 hours, 72 hours and 96 hours. After 96 hours of exposure to JAK inhibitors, cells were washed in normal culture medium, to remove momelotinib and ruxolitinib. Subsequently, half of the cells were incubated with 1.5 µM momelotinib or 0.75 µM ruxolitinib for another 4 hours, or 24 hours, whereas the remaining cells were incubated in vehicle control (Ctr) medium. Protein expression levels were examined using western blot (25 µg lysate). Expression levels of phospho-proteins were quantified relative to total protein.

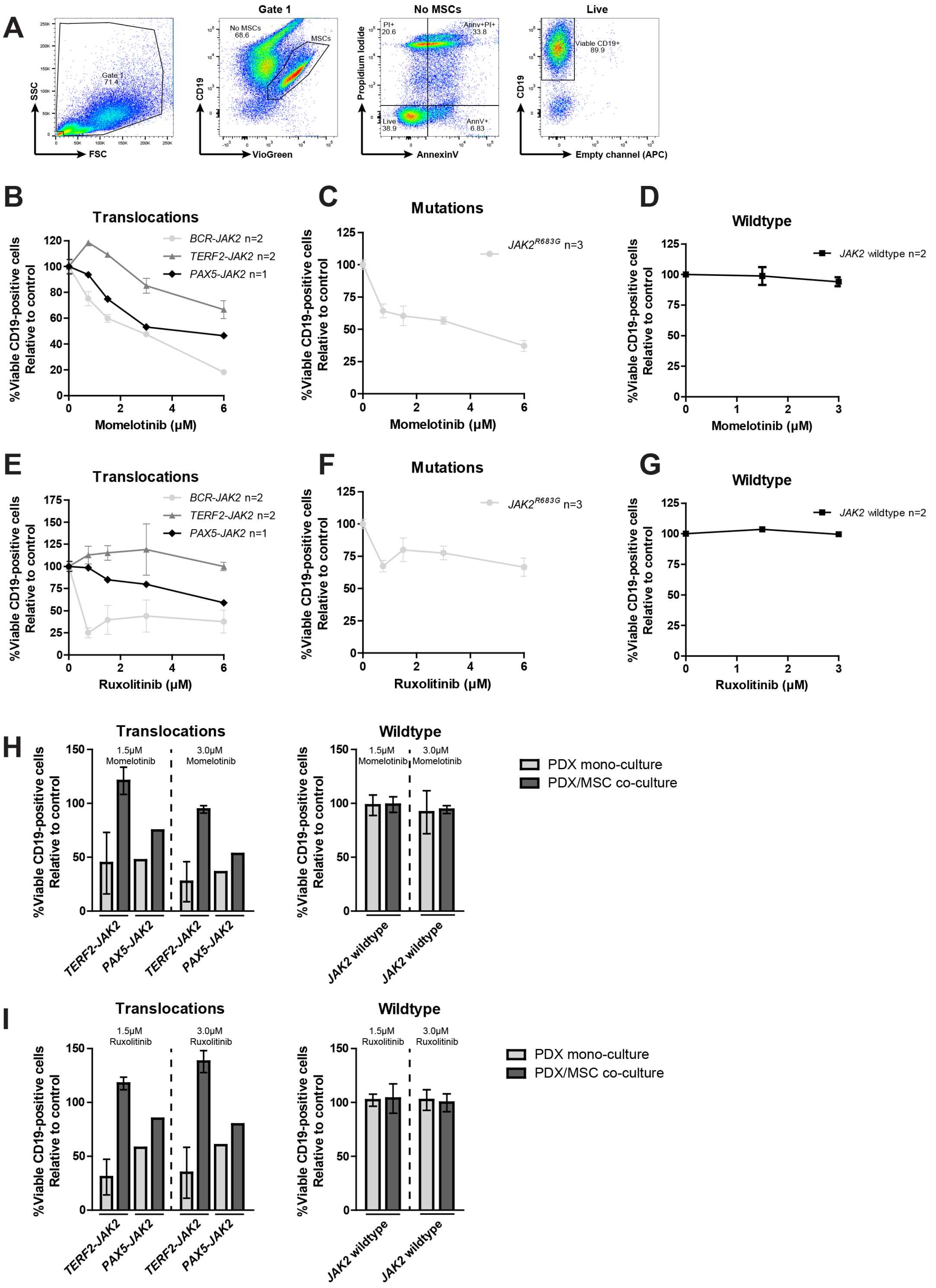

Supplementary Figure 13: The efficacy of JAK inhibitors in PDX/MSC co-cultures. The response of PDX cells (CD19+) co-cultured with MSCs (CD19-) to increasing concentrations of momelotinib and ruxolitinib was assessed after four days of culture using flow cytometry. Viability was calculated relative to untreated controls. Mean±SEM is shown. **(A)** Gating strategy. **(B-G)** Percentage of viable PDX cells being JAK2 translocated (*BCR-JAK2* n=2; *TERF2-JAK2* n=2; *PAX5-JAK2* n=1), *JAK2* mutated (*JAK2*^{R683G} n=3), or *JAK2* wildtype cells (*JAK2* wildtype n=2), co-cultured with patient MSCs, after four days exposure to momelotinib and ruxolitinib. **(H-I)** The effect of 1.5 µM and 3.0 µM momelotinib or ruxolitinib on the viability of *JAK2* translocated PDX cells in mono-culture, or in co-culture with patient MSCs. *TERF2-JAK2* n=2; *PAX5-JAK2* n=1, JAK2 wildtype n=2.

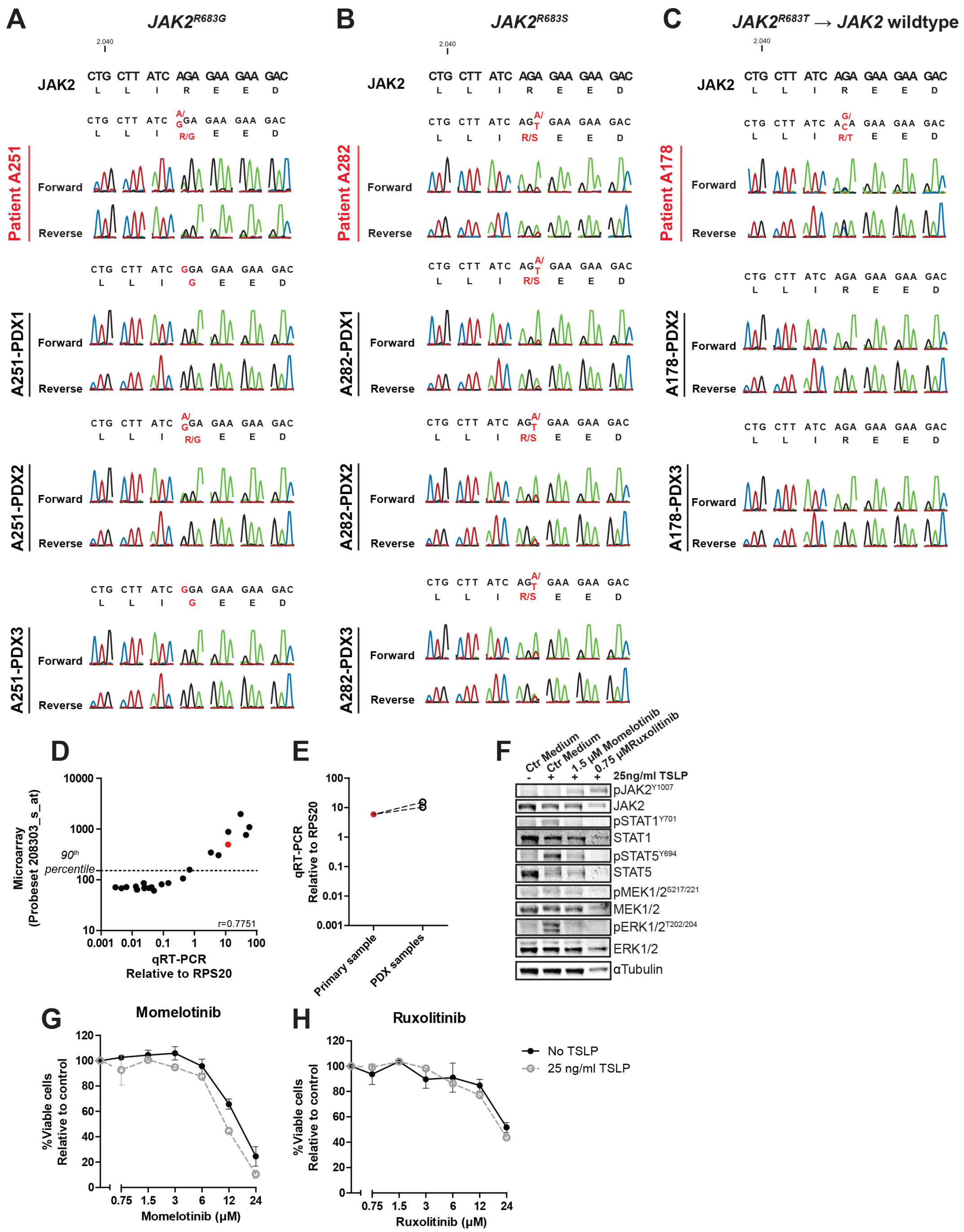

Supplementary Figure 14: JAK2 mutations in primary leukemic and PDX cells. **(A-C)** Primary patient cells (A251, A282, and A178) were injected intra-femoral into three NSG mice per patient. After engraftment, leukemic cells were harvested from these mice (PDX1, PDX2, and PDX3). Genomic DNA was used to identify *JAK2* mutations in exon 16 (forward primer 5'-ATGCCTCCAAATTATTATACTATCA-3', reverse primer 5'-ATCACCTCACAGTCCA TGGTTAT-3') in these PDX cells and primary leukemic cells, using Sanger sequencing. **(D)** *CRLF2* expression in primary leukemic samples was determined using qRT-PCR and SYBR green. qRT-PCR expression values were correlated to Affymetrix microarray data of probeset 208303_s_at. Spearman correlation coefficient is reported. **(E)** *CRLF2* expression levels in the primary leukemic and PDX samples was determined using qRT-PCR. *CRLF2* expression was calculated relative to *RSP20* expression levels. **(F)** *JAK2* wildtype cells were incubated for 1h with 25 ng/ml TSLP. Effect of TSLP on protein expression levels was examined using western blotting. **(G-H)** *JAK2* wildtype cells were exposed for four days to indicated concentrations of momelotinib or ruxolitinib. Viability was measured using an MTT assay, after which cell survival was calculated relative to vehicle treated controls. Mean±SD of duplicates are shown.

Supplementary Table 1: RT-PCR primers used for the detection of *JAK2* translocations

Gene fusion	Forward primer ID	Forward sequence 5′ to 3′	Reverse primer ID	Reverse sequence 5′ to 3′
**ATF7IP-JAK2**	ATF7IP_ exon12_F2	aacccatacaaccagcaccgcctct	JAK2_ exon20_R4	tgttgtcatgctgtagggatttcagga
**BCR-JAK2**	BCR_ exon1_F1	gtgccataagcggcaccggcact	JAK2_ exon18_R1	aggcctgaaatctggttcata
**EBF1-JAK2**	EBF_ exon14_F2	cacgagcatgaacggatacggctct	JAK2_ exon20_R4	tgttgtcatgctgtagggatttcagga
**ETV6-JAK2**	ETV6_ exon3_F1	atggcaaagctctcctgctgctgac	JAK2_ exon20_R4	tgttgtcatgctgtagggatttcagga
**PAX5-JAK2**	PAX5_ exon3_F2	acaatgacaccgtgcctagcgtcag	JAK2_ exon19_	tcaaaggcaccagaaaac
**PPFIBP1-JAK2**	PPFIBP1_ exon10_F1	tgcaagatgaaaggagaaggggttga	JAK2_ exon20_R4	tgttgtcatgctgtagggatttcagga
**SSBP2-JAK2**	SSBP2_ exon7_F1	ggcacttggaggtgtcccaggaagt	JAK2_ exon20_R4	tgttgtcatgctgtagggatttcagga
**STRN3-JAK2**	STRN3_ exon7_F3	tgaaggagctggagaagcacggagt	JAK2_ exon20_R4	tgttgtcatgctgtagggatttcagga
**TPR-JAK2**	TPR_ exon38_F1	tggaaatgcctcttccaaagaagttga	JAK2_ exon20_R4	tgttgtcatgctgtagggatttcagga
**TERF2-JAK2**	TERF2_ exon10_F1	tggggaaggaaactgg	JAK2_ exon20_R4	tgttgtcatgctgtagggatttcagga

PCR program

50°C	2 minutes	
95°C	10 minutes	
95°C	15 seconds	8 cycles
60°C	1 minute	
72°C	30 seconds	
95°C	15 seconds	8 cycles
57°C	1 minute	
72°C	30 seconds	
95°C	15 seconds	8 cycles
54°C	1 minute	
72°C	30 seconds	
95°C	15 seconds	8 cycles
51°C	1 minute	
72°C	30 seconds	
95°C	15 seconds	8 cycles
48°C	1 minute	
72°C	30 seconds	
